# Performance evaluation for MOTIFSIM

**DOI:** 10.1186/s12575-018-0088-3

**Published:** 2018-12-18

**Authors:** Ngoc Tam L. Tran, Chun-Hsi Huang

**Affiliations:** Department of Computer Science and Engineering, University of Connecticut, Storrs, CT 06269 USA

**Keywords:** Binding sites, DNA motif, Motif detection tool, Motif similarity comparison, Motif clustering, Merging similar motifs

## Abstract

**Background:**

Previous studies show various results obtained from different motif finders for an identical dataset. This is largely due to the fact that these tools use different strategies and possess unique features for discovering the motifs. Hence, using multiple tools and methods has been suggested because the motifs commonly reported by them are more likely to be biologically significant.

**Results:**

The common significant motifs from multiple tools can be obtained by using MOTIFSIM tool. In this work, we evaluated the performance of MOTIFSIM in three aspects. First, we compared the pair-wise comparison technique of MOTIFSIM with the un-gapped Smith-Waterman algorithm and four common distance metrics: average Kullback-Leibler, average log-likelihood ratio, Chi-Square distance, and Pearson Correlation Coefficient. Second, we compared the performance of MOTIFSIM with RSAT Matrix-clustering tool for motif clustering. Lastly, we evaluated the performances of nineteen motif finders and the reliability of MOTIFSIM for identifying the common significant motifs from multiple tools.

**Conclusions:**

The pair-wise comparison results reveal that MOTIFSIM attains better performance than the un-gapped Smith-Waterman algorithm and four distance metrics. The clustering results also demonstrate that MOTIFSIM achieves similar or even better performance than RSAT Matrix-clustering. Furthermore, the findings indicate if the motif detection does not require a special tool for detecting a specific type of motif then using multiple motif finders and combining with MOTIFSIM for obtaining the common significant motifs, it improved the results for DNA motif detection.

**Electronic supplementary material:**

The online version of this article (10.1186/s12575-018-0088-3) contains supplementary material, which is available to authorized users.

## Background

Transcription factors (TFs) are proteins that can bind to several regions of DNA. The binding regions are short sequences of DNA called transcription factor binding sites (TFBSs). They typically range from 8-10 to 16–20 bp [1]. The TFs bind to DNA in a particular way that the binding sites are similar and they differ only by some nucleotides from one another [1]. Several similar binding sites form a binding site motif. The binding between TFs and DNA has an important role in gene expression as it controls several vital processes in development, responses to environmental stresses, diseases, and many others [2]. Detecting binding site motifs can reveal the TFs that control the gene expression. Thus, numerous motif finders have been developed such as MEME [3], DREME [4], MEME-ChIP [5], CisFinder [6], RSAT peak-motifs [7], PScanChIP [8], and W-ChIPMotifs [9] among many others. We reviewed nine Web tools for finding binding site motifs in ChIP-Seq data [10]. The results reveal that different tools reported different results for an identical dataset. The cause is that they implemented different algorithms and possess unique features for discovering the motifs. Hence, using multiple tools and methods has been advised because the motifs commonly reported by them are more likely to be biologically significant [10]. Nevertheless, the results from multiple tools need to be compared for identifying the common significant motifs. MOTIFSIM tool was designed for this purpose in our previous studies [11, 12].

In this work, we evaluated the performance of MOTIFSIM in three aspects. First, we compared the pair-wise comparison technique of MOTIFSIM with the un-gapped Smith-Waterman (USW) algorithm [13] and four common distance metrics namely average Kullback-Leibler (AKL) [14], average log-likelihood ratio (ALLR) [15], Chi-Square distance (CS) [16], and Pearson Correlation Coefficient (PCC) [17]. Second, we compared MOTIFSIM with RSAT matrix-clustering tool for motif clustering [18]. Finally, we assessed the performances of nineteen motif finders and the reliability of MOTIFSIM for identifying the common significant motifs from multiple tools.

## Methods

The reader can find the original MOTIFSIM algorithm in the Additional file 1. A detailed discussion of this algorithm can be found in [11] and a slightly modified version of it can also be found in [12].

### Assessing MOTIFSIM algorithm for pair-wise motif comparison

We evaluated MOTIFSIM for both string-based and matrix-based pair-wise comparisons. For string-based comparison, we compared MOTIFSIM with the USW algorithm. The motifs are in IUPAC string format [19]. We implemented the *NUC.4.4* substitution matrix for this comparison [20]. We chose USW as it has been studied by Mahony et al. for motif similarity comparison and the authors showed it is more efficient when it is used with other column metric [21]. For matrix-based comparison, we assume the columns are independent in the matrices. We compared MOTIFSIM with AKL, ALLR, CS, and PCC. These distance metrics have been used in several studies for measuring similarity between motifs [16, 21–24]. We used a minimum overlapping window of four columns for pair-wise comparisons as presented in [11]. For each overlapping position between two matrices in both forward and backward directions including their reverse complements, we calculated a similarity score between them by using AKL, ALLR, CS, PCC, and MOTIFSIM.

#### Un-gapped Smith-Waterman algorithm

The Smith-Waterman (SW) algorithm is a local pair-wise sequence alignment for finding the local regions that have highest similarity between two sequences. In this assessment, we did not allow gaps for local alignment. The USW pair-wise sequence alignment returns a best raw score *S*. To obtain the statistical significance for this raw score, we calculated the expected number of un-gapped alignments with score *S* found with random sequences by using eq. (1) [25].

1 $$ E(S)= Kmn{e}^{-\lambda S} $$

where *S* is a raw score of the alignment, *m* and *n* are the lengths of two sequences, *K* and *λ* are Karlin-Altschult statistics parameters, and *E* is the *E*-value of the score *S*. *BLAST* uses *K* = 0.132 and *λ* = 0.316 [26, 27]. In this evaluation, we used *K* = 0.1 and *λ* = 0.3. Since we compared a given motif with several other motifs, we selected the smallest *E*-value for determining the best match for a given motif. This *E*-value is the expected number of sequences that produce the same or better score by chance. To perform a pair-wise comparison using MOTIFSIM, we used a similarity threshold of 75% or more. This threshold has been evaluated in our previous study and showed to be efficient for comparison [11].

#### Distance metrics

Table 1 shows four distance metrics for comparing with MOTIFSIM. The AKL is the weighted average of log-likelihood ratio distance between two distributions [21]. We adopted it from Mahony et al. [21]. The authors subtract the AKL score from 10 to convert it into a similarity score. The ALLR was adopted from Schones et al. [24]. It is a weighted sum of two log-likelihood ratios that was introduced by Wang and Stormo [24]. We used a prior probability of 0.25 for base *b* for this distance metric. We also implemented the PCC from Schones et al. [24]. The PCC is a popular metric for measuring the correlation between two sets of variables. In this case, they are two aligned columns of two matrices. We calculated the score for an alignment position between two matrices by taking the sum of individual column comparison scores for three distance metrics above. We adopted the Fisher-Irwin exact test that was used by Schones et al. [24] for calculating the *P*-value of a similarity score obtained at an alignment position of two columns *X* and *Y*. The *P*-value for an alignment position between two matrices is the product of *P*-values of the individual columns [24]. We used a *P*-value ≤0.05 for filtering out the insignificant scores as they indicate a significant dissimilarity between two matrices. Thus, a larger *P*-value indicates more similar between two matrices. We selected the largest *P*-value to represent the best alignment between two matrices.

**Table 1:** Four distance metrics used in pair-wise comparisons with MOTIFSIM

Metric	Formula	Description	Ref.
Average Kullback-Leibler (AKL)	$AKL\left(X,Y\right)=10-\frac{\sum \limits_{b=A}^T{f}_x(b)\times \mathit{\log}\frac{f_x(b)}{f_y(b)}+\sum \limits_{b=A}^T{f}_y(b)\times \mathit{\log}\frac{f_y(b)}{f_x(b)}}{2}$	*X* and *Y* are two aligned columns of two matrices in comparison. *f*_*x*_(*b*) is the frequency of base *b* ∈ {*A*, *C*, *G*, *T*} in column *X* and likewise for *f*_*y*_(*b*) in column *Y.* *AKL*(*X*, *Y*) is the similarity score at an alignment position for two columns *X* and *Y*.	21
Average Log-likelihood Ratio (ALLR)	$ALLR=\frac{\sum \limits_{b=A}^T{n}_{bX}\times \mathit{\log}\left(\frac{f_{bY}}{p_b}\right)+\sum \limits_{b=A}^T{n}_{bY}\times \mathit{\log}\left(\frac{f_{bX}}{p_b}\right)}{\sum \limits_{b=A}^T\left({n}_{bX}+{n}_{bY}\right)}$	*n*_*bX*_ is the count of base *b* ∈ {*A*, *C*, *G*, *T*} in column *X* and likewise for *n*_*bY*_ in column *Y*. *f*_*b*_ = *n*_*b*_/*N* is the frequency of base *b* where *N* is the total count of all bases in a column. *p*_*b*_ is the prior probability for base *b*.	24
Pearson Correlation Coefficient (PCC)	$PCC\left(X,Y\right)=\frac{\sum \limits_{b=A}^T\left({X}_b-\overline{X}\right)\times \left({Y}_b-\overline{Y}\right)}{\sqrt{\sum \limits_{b=A}^T{\left({X}_b-\overline{X}\right)}^2\times \sum \limits_{b=A}^T{\left({Y}_b-\overline{Y}\right)}^2}}$	*X*_*b*_ is the count of base *b* ∈ {*A*, *C*, *G*, *T*} in column *X* and likewise for *Y*_*b*_ in column *Y*. $\overline{X}$ is the average count of bases in column *X* and likewise for $\overline{Y}$ in column *Y*.	24
χ^2^ Distance	${\chi}^2=\sum \limits_{b=A,C,G,T}\frac{{\left({N}_{g,i}{f}_{b,i}-{N}_{f,i}{g}_{b,i}\right)}^2}{N_{f,i}{N}_{g,i}\left({f}_{b,i}+{g}_{b,i}\right)}$	*f*_*b*, *i*_ is the entries of overlapping parts at position *i* in matrix *f* of the two matrices *f* and *g* in comparison *g*_*b*, *i*_ is the entries of overlapping parts in matrix *g* *N*_*f*, *i*_ = ∑_*b*_*f*_*b*, *i*_, and *N*_*g*, *i*_ = ∑_*b*_*g*_*b*, *i*_.	16

Lastly, we adopted the χ^2^ distance from Kielbasa et al. for comparing with MOTIFSIM [16]. It is also a popular metric for measuring the distance between position frequency matrices. We calculated the *χ*^2^ distance for the aligned columns at position *i* by using the equation in Table 1. We used a threshold ≤7.81, which corresponds to a *P*-value ≤0.05 for selecting a significant similarity score at each position [16]. The distance *D* between two matrices is obtained by counting the number of *χ*^2^ scores that exceed the threshold of 7.81 in the alignment of two matrices [16]. Thus, a smaller *D* value represents a better match between two motifs. We selected the smallest *D* among all possible alignments between two motifs to represent the best score between them.

### MOTIFSIM

The core of MOTIFSIM algorithm is pair-wise alignments of position specific probability matrices (PSPMs). The similarity score of an alignment can be selected by using the percentage. In our previous study [11], it showed a 75% or more to be an efficient threshold for filtering the motifs. Hence, we used this threshold here again for comparisons.

### Motif clustering comparison

The core of Matrix-clustering is pair-wise comparisons of Position Specific Scoring Matrices. The similarity between motifs is measured by using RSAT compare-matrices, which allows combining several distance metrics for similarity calculation [18]. The tool builds a global hierarchical tree from bottom up by using the similarity scores calculated from pair-wise comparisons [18]. MOTIFSIM also performs pair-wise comparisons on PSPMs. The similarity scores calculated by MOTIFSIM are used to build the distance matrices, which are fed into *hclust* function in *R* for building the trees [12]. The *hclust* function also implemented the hierarchical clustering algorithm.

We compared the performances of both tools for clustering the motifs that were selected from the Jaspar database [28]. The method for selecting the motifs is presented in the Datasets section. We used the default setting provided by each tool to run the experiments. The results were generated in multiple formats including tree format for comparisons. We obtained the count for the motifs that were correctly classified into their family in the database by each tool for each dataset. A family can have at least two or more members. The count was then used for calculating the percentage of correct classification by each tool.

### Measuring the significance of the global significant motif

We used the assessment method, the benchmark sequence datasets, and the on-line assessment tool from Tompa et al. for this evaluation [29]. We measured the performances of nineteen motif finders on various benchmark sequence datasets [29]. For each tool *T* and each dataset *D*, we have a set of known binding sites and a set of predicted binding sites. Thus, the performance of *T* on *D* can be measured at *nucleotide level* and at *site level* [29]. At the nucleotide level, we calculated four statistics namely sensitivity (*nSn*), positive predictive value (*nPPV*), specificity (*nSP*), and correlation coefficient (*nCC*). Similarly, at site level, we calculated two statistics that are sensitivity (*sSn*) and positive predictive value (*sPPV*). These statistics are presented in the Additional file 1 [29].

The motifs generated by various tools for an identical sequence dataset were fed into MOTIFSIM for generating the global significant motifs [11]. Since MOTIFSIM identifies a list of common significant motifs from a pool of motifs reported by various tools, we selected the best common significant motif based on two criteria. First, it must represent the popular vote by the majority of the tools. Second, it has the highest rank of similarity score. Since we know the origin of the common significant motif, its significance can be calculated by using six statistics above. We assessed the correctness of the motif reported by each tool and this assessment covers the selected motif from MOTIFSIM. We then compared the correctness for identifying the known motif of each tool including MOTIFSIM.

### Datasets

The motif datasets that were used in the assessment came from sixteen benchmark sequence datasets in Table 2 [29]. The sequence datasets came from three species: *Homo sapiens*, *Mus musculus*, and *Saccharomyces cerevisiae*. The sequence datasets can be in *generic* or *Markov* type [29]. The generic type was generated by randomly selecting the promoter sequences and then implanted the known binding sites of the same species into those sequences. The Markov type was created by generating random sequences using Markov chain order of 3 and then implanted the known binding sites of the same species into those sequences. Each known binding site embedded in a sequence belongs to a specific transcription factor in the TRANSFAC database [30]. We selected different benchmark datasets so that each sequence in a dataset contains at least one or more embedded sequences of the same transcription factor. These sequence datasets were used to run nineteen motif finders [3, 29, 31–35] in Table 3 for generating the motifs that were subsequently used in this assessment. Some general characteristics of these tools can also be found in Additional file 1: Table S1. In addition, we selected 46 motifs from the TRANSFAC database. Each selected motif has at least one or more closely structural members of the same species in the database. The aim is to measure the performances of USW, AKL, ALLR, PCC, CS, and MOTIFSIM for identifying the known motif among several similar motifs of the same species in the TRANSFAC database. The performance of each method is measured by using the number of motifs that were correctly identified by each method for the same set of datasets.

**Table 2:** Sixteen benchmark sequence datasets [29]. They are grouped by species. Each sequence dataset has an embedded transcription factor

Sequence Dataset	Dataset Type	Species	Transcription Factor	Number of Sequences	Sequence Length
hm01g	Generic	*Homo sapiens*	AP-1	18	2000
hm04g	Generic	*Homo sapiens*	c-Jun	13	2000
hm08m	Markov	*Homo sapiens*	CREB	15	500
hm15g	Generic	*Homo sapiens*	NF-1	4	2000
hm17g	Generic	*Homo sapiens*	NF-kappaB	11	500
hm19g	Generic	*Homo sapiens*	Sp1	5	500
hm22g	Generic	*Homo sapiens*	USF1	6	500
hm22m	Markov	*Homo sapiens*	USF1	6	500
mus04m	Markov	*Mus musculus*	C/Ebalpha	7	1000
mus06g	Generic	*Mus musculus*	GATA-1	3	500
mus10g	Generic	*Mus musculus*	Sp1	13	1000
mus11m	Markov	*Mus musculus*	Sp1	12	500
yst02g	Generic	*Saccharomyces cerevisiae*	GAL04	4	500
yst03m	Markov	*Saccharomyces cerevisiae*	GCN4	8	500
yst06g	Generic	*Saccharomyces cerevisiae*	MCM1	7	500
yst09g	Generic	*Saccharomyces cerevisiae*	CAR1	16	1000

**Table 3:** Nineteen motif finders used in the assessment. An x mark associates a sequence dataset with a tool. The sequence datasets are grouped by species. Thirteen tools in *italic* face are older tools used by Tompa et al. [29]. The rest are newer tools. The datasets with (*) were used to run newer tools

	Datasets																
	Homo Sapiens								Mus Musculus				Saccharomyces Cerevisiae				
Tool Name	hm01g*	hm04g*	hm08m	hm15g*	hm17g*	hm19g*	hm22g*	hm22m	mus04m	mus06g	mus10g	mus11m	yst02g	yst03m	yst06g	yst09g*	Ref.
*AlignACE*			x						x	x	x	x		x			29
*ANN-Spec*			x					x	x	x	x	x	x	x	x		29
ChIPMunk	x	x		x	x	x	x									x	31
*Consensus*									x	x	x	x		x			29
DMINDA	x	x		x	x	x	x									x	32
*GLAM*			x					x	x	x	x	x	x	x	x		29
*Improbizer*			x					x	x	x	x	x	x	x	x		29
*MEME (older version)*			x					x	x	x		x	x	x	x		29
MEME (v. 4.11.4)	x	x		x	x												3
*MITRA*			x					x	x	x	x	x	x	x	x		29
*MotifSampler*			x					x	x	x		x	x	x	x		29
*oligodyad-analysis*			x					x	x	x	x		x	x	x		29
peak-motifs	x	x		x	x	x	x									x	33
*QuickScore*			x							x	x		x	x	x		29
*SeSiMCMC*			x					x	x	x	x	x	x	x	x		29
STEME	x	x		x			x										34
*Weeder*			x					x	x	x	x	x	x	x	x		29
XXmotif	x	x		x	x												35
*YMF*			x					x	x	x	x	x	x	x	x		29

The data that were used in the first phase of the assessment contain 158 motifs. They can be found in Additional file 1: Table S2. The first one hundred and four are on-line predicted motifs, which were generated by thirteen tools in Tompa et al. [29]. Since thirteen tools in [29] are older tools, we assessed six additional newer tools in Table 3. Because nine sequence datasets used by Tompa et al. to run older tools produced low performance results in their study, we selected seven additional sequence datasets to run the newer tools. They are marked with asterisk (*) in Table 3. The objective was to observe if a sequence dataset had any influence on the performance of each tool. The next eight motifs in the collection came from five newer tools that are ChIPMunk [31], DMINDA [32], MEME (v. 4.11.4) [3], peak-motifs [33], and XXmotif [35]. We followed the procedure suggested by Tompa et al. for selecting the top three motifs for each sequence dataset and we calculated six statistics above for each motif. We used *nCC* for selecting the best motif reported by each tool for each sequence dataset. The following forty-six motifs in the collection came from the TRANSFAC database. For each motif in the collection, we performed pair-wise comparisons with motifs of the same species in the TRANSFAC database by using USW, AKL, ALLR, PCC, CS, and MOTIFSIM. The second phase of the assessment used four datasets containing the motifs selected from the Jaspar database [28]. They can be found in Table 4. The datasets came from four taxonomic groups namely *Fungi*, *Insects*, *Plants* and *Vertebrates*. Each dataset comprises motifs from different families. The goal was to cluster them into a proper family, which they belong in the Jaspar database. The details of each dataset can be found in Additional file 1: Tables S3-S6.

**Table 4:** Four datasets used in motif clustering comparisons. The motifs in each dataset were selected from the Jaspar database [28]

Dataset	Number of Motifs	Taxonomic Group
pfm_fungi	78	Fungi
pfm_insect	42	Insects
pfm_plant	65	Plants
pfm_vertebrate	73	Vertebrates

Lastly, the data that were used in the third phase of the assessment include 137 motifs. They can be found in Additional file 1: Table S7. The first thirty-three are predicted motifs, which were generated by six newer tools. The rest are predicted motifs generated by thirteen older tools.

## Results

### Pair-wise motif comparison

We obtained the number of motifs that were correctly identified by each method per sequence dataset for 112 predicted motifs in the collection. Subsequently, we calculated the percentage of motifs that were correctly identified by each method. MOTIFSIM attains 31% comparing to 22% for USW, 1% for AKL, 0% for ALLR, 0% for PCC, and 15% for CS as shown in Table 5.

**Table 5:** Performance comparisons for USW, AKL, ALLR, PCC, CS, and MOTIFSIM for the predicted motifs in the collection. The number of motifs that were correctly identified by each method per sequence dataset is listed. The percentage of motifs that were correctly identified by each method per dataset was also calculated

Sequence Dataset	Number of Motifs Correctly Identified							% of Motifs Correctly Identified					
	USW	AKL	ALLR	PCC	CS	MOTIFSIM	Total # of Tools	USW	AKL	ALLR	PCC	CS	MOTIFSIM
hm08m	0	0	0	0	0	1	12	0%	0%	0%	0%	0%	8%
hm17g	2	0	0	0	3	2	5	40%	0%	0%	0%	60%	40%
hm22m	1	0	0	0	2	1	10	10%	0%	0%	0%	20%	10%
mus04m	0	0	0	0	0	2	12	0%	0%	0%	0%	0%	17%
mus06g	1	1	0	0	1	2	13	8%	8%	0%	0%	8%	15%
mus10g	3	0	0	0	0	5	11	27%	0%	0%	0%	0%	45%
mus11m	2	0	0	0	0	3	11	18%	0%	0%	0%	0%	27%
yst02g	6	0	0	0	7	6	11	55%	0%	0%	0%	64%	55%
yst03m	3	0	0	0	1	9	13	23%	0%	0%	0%	8%	69%
yst06g	5	0	0	0	2	3	11	45%	0%	0%	0%	18%	27%
yst09g	2	0	0	0	1	1	3	67%	0%	0%	0%	33%	33%
Total	25	1	0	0	17	35	112	22%	1%	0%	0%	15%	31%

We repeated the calculations above but for the selected motifs from the TRANSFAC database in the collection. We also obtained the number of motifs that were correctly identified by each method per species as shown in Table 6. Again, we calculated the percentage of motifs that were correctly identified by each method. MOTIFSIM attains 98% comparing to 61% for USW, 100% for AKL, 100% for ALLR, 100% for PCC, and 85% for CS. Although MOTIFSIM has a slightly lower percentage than AKL, ALLR, and PCC for this portion of comparison, the average percentage for both comparisons demonstrates it has higher overall performance than other methods. Specifically, MOTIFSIM attains 64.5% comparing to 41.5% for USW, 50.5% for AKL, 50% for ALLR, 50% for PCC, and 50% for CS as shown in Table 7. In general, different methods exhibit various performances on different datasets. However, the overall results show MOTIFSIM outperforms other methods.

**Table 6:** Performance comparisons for USW, AKL, ALLR, PCC, CS, and MOTIFSIM for the selected motifs from TRANSFAC database in the collection. The number of motifs that were correctly identified by each method per species is listed. The percentage of motifs that were correctly identified by each method per species was also calculated

	Number of Motifs Correctly Identified							% of Motifs Correctly Identified					
Species	USW	AKL	ALLR	PCC	CS	MOTIFSIM	Total # of Motifs by Species	USW	AKL	ALLR	PCC	CS	MOTIFSIM
*Homo sapiens*	11	19	19	19	17	19	19	58%	100%	100%	100%	89%	100%
*Mus musculus*	7	15	15	15	14	14	15	47%	100%	100%	100%	93%	93%
*Saccharomyces cerevisiae*	4	5	5	5	4	5	5	80%	100%	100%	100%	80%	100%
*Drosophila melanogaster*	6	7	7	7	4	7	7	86%	100%	100%	100%	57%	100%
Total	28	46	46	46	39	45	46	61%	100%	100%	100%	85%	98%

**Table 7:** Average percentage for the predicted motifs and the selected motifs by each method. MOTIFSIM achieves higher performance than other methods

% of Motifs Correctly Identified						
Motif Category	USW	AKL	ALLR	PCC	CS	MOTIFSIM
Predicted motifs	22%	1%	0%	0%	15%	31%
Selected motifs from TRANSFAC	61%	100%	100%	100%	85%	98%
Average percentage	41.5%	50.5%	50%	50%	50%	64.5%

### Motif clustering

**Fig. 1:**
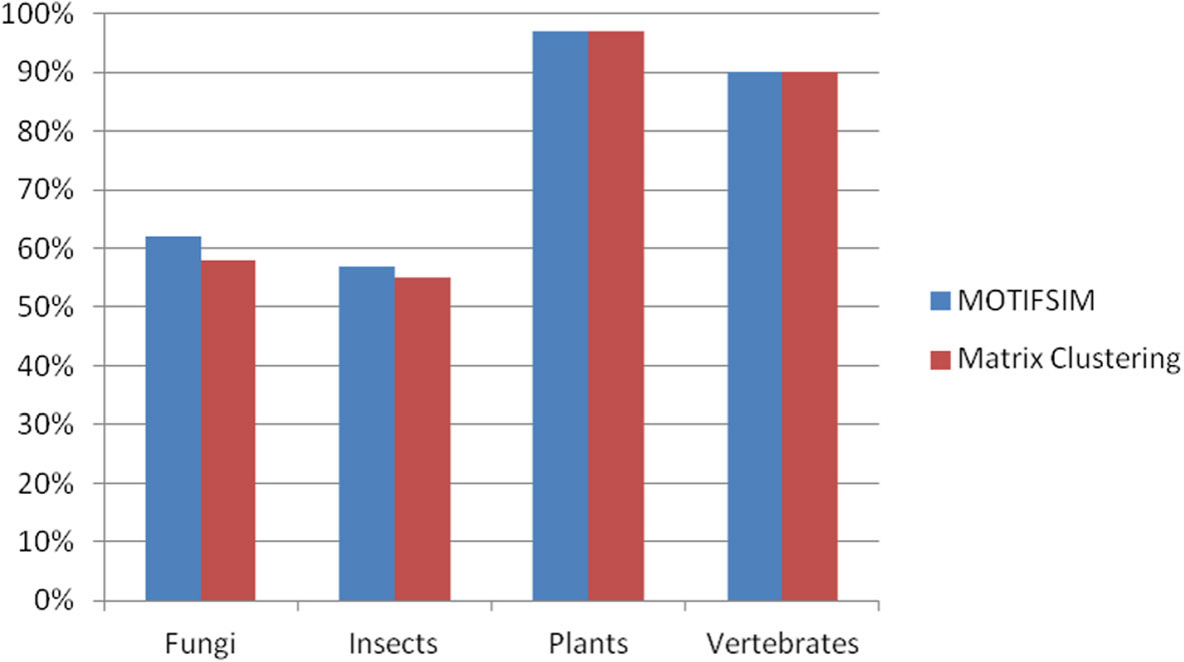
Performance comparison for MOTIFSIM and RSAT Matrix-clustering tool on four taxonomic datasets: Fungi, Insects, Plants, and Vertebrates. MOTIFSIM has higher accurate percentages than Matrix-clustering for Fungi and Insects datasets. It achieves 62% for Fungi and 57% for Insects datasets comparing to 58% and 55% respectively from Matrix-clustering. For Plants and Vertebrates datasets, both tools achieve similar accurate percentages with 97% and 90% respectively

To compare the performances of MOTIFSIM and Matrix-clustering, we obtained the motif tree for the result generated by each tool for each dataset. We used the Phylodendron tool to generate the motif trees for the results from Matrix-clustering [36]. The trees are shown in Additional file 1: Figures S1-S8. We calculated the percentage of motifs that were correctly classified into their family by each tool per dataset. MOTIFSIM achieves 62% for *Fungi* and 57% for *Insects* datasets comparing to 58% and 55% respectively from Matrix-clustering. For the *Plants* and *Vertebrates* datasets, both tools achieve similar results of 97% and 90% respectively. The comparison results are in Table 8 and Fig. 1.

**Table 8:** Comparison results for Matrix-clustering and MOTIFSIM for four taxonomic datasets. The number of motifs that were correctly classified and the percentage of correct classification by each tool for each dataset are shown. MOTIFSIM has a similar or better performance than Matrix-clustering

Dataset	Total Number of Motifs	MOTIFSIM		Matrix Clustering	
		# of Motifs Correctly Clustered	% of Correct Classification	# of Motifs Correctly Clustered	% of Correct Classification
Fungi	78	48	62%	45	58%
Insects	42	24	57%	23	55%
Plants	65	63	97%	63	97%
Vertebrates	73	66	90%	66	90%

### Significance of the global significant motif

**Fig. 2:**
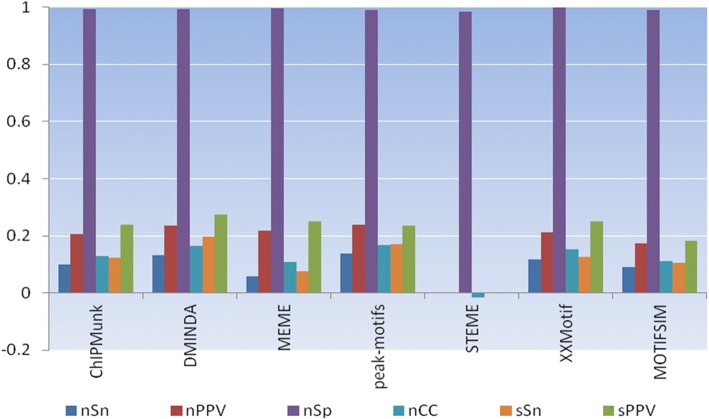
Average statistics for six newer motif finders (ChIPMunk, DMINDA, MEME v. 4.11.4, peak-motifs, STEME, XXmotif) and MOTIFSIM on seven additional sequence datasets. The first four statistics at the bottom of the figure are nucleotide level statistics. The next two are site level statistics. STEME shows lower performance than all other tools due to its nature design and implementation. MOTIFSIM has better performance than MEME and STEME and it is in an intermediate range comparing to other tools

**Fig. 3:**
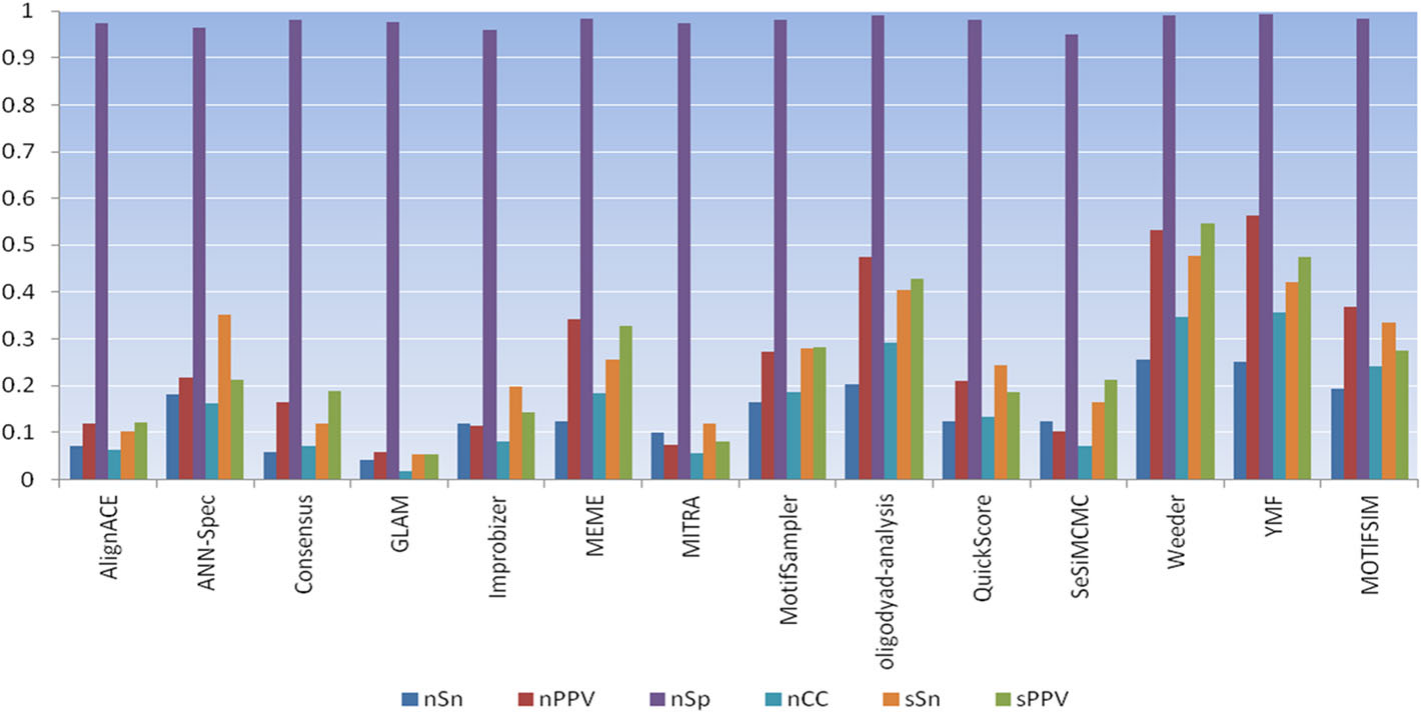
Average statistics for thirteen older motif finders and MOTIFSIM on nine sequence datasets. The older tools and nine sequence datasets were used by Tompa et al. in their study [29]. The first four statistics at the bottom of the figure are nucleotide level statistics. The next two are site level statistics. MOTIFSIM attains better performance than ten other tools except for Oligodyad-analysis, Weeder, and YMF

We measured the performances of all tools including MOTIFSIM by calculating six statistics presented above for the best motif produced by each tool for the same sequence dataset. Since the selected global significant motif from MOTIFSIM came from one of the motif finders, its correctness can be measured by using six statistics above. The results of different tools including MOTIFSIM for each sequence dataset are in Additional file 1: Figures S9-S24. Additional file 1: Figures S9-S11 show the results for six newer tools including MOTIFSIM for the sequence datasets *hm01g*, *hm04g*, and *hm15g* respectively. In Additional file 1: Figure S9, the selected global significant motif from MOTIFSIM came from peak-motifs. This tool has a better performance than other tools. Additional file 1: Figures S10 and S11 show seven tools failed to identify the known motif. However, Additional file 1: Figure S12 indicates all five newer tools and MOTIFSIM successfully identified the known motif for the sequence dataset *hm17g*. The selected global significant motif from MOTIFSIM came from peak-motifs. STEME was absent in this figure because it did not report any significant motif. Additional file 1: Figures S13-S15 show the results for three or four newer tools including MOTIFSIM for the sequence datasets *hm19g*, *hm22g*, and *yst09g* respectively. Other newer tools were absent in these figures because they did not report any significant motif. The results for older tools including MOTIFSIM are shown in Additional file 1: Figures S16-S24. In Additional file 1: Figure S16, the selected global significant motif from MOTIFSIM came from YMF. This tool has a better performance than some other tools. Generally, some tools exhibit better performance than others for some sequence datasets. We calculated the average statistics for six newer tools including MOTIFSIM. The result reveals STEME has a poorer performance than other tools as shown in Fig. 2. We also calculated the average statistics for thirteen older tools including MOTIFSIM. The result in Fig. 3 indicates Weeder, YMF, and Oligodyad-analysis attain better performance than other tools. MOTIFSIM is in an intermediate range comparing to Weeder and YMF. However, it achieves better performance than ten other tools except for Oligodyad-analysis, Weeder, and YMF.

## Discussion

Using multiple tools for finding motifs is generally advised because the motifs reported by multiple tools are more likely to be biologically significant. In this assessment, the predicted motif was not verified with the known motif for the objective of measuring the performance of each tool. In general, the results show that some tools have better performance than others. Some tools show poor performance and some even failed to identify the known motif. However, the observation for Fig. 2 indicates the top two performers: peak-motifs and DMINDA outperform other tools while MEME and STEME exhibit lower performance than others with STEME is at the lowest rank. Since each tool has its unique approach for detecting the motifs, the method that each tool used generally falls into one of the two common categories: profile-based method and consensus-based method. We observed the type of method that each tool is based on in Additional file 1: Table S1. DMINDA is a graph-based method and peak-motifs is a word-based method, which is a subcategory of the consensus-based method. Both MEME and STEME are profile-based methods. However, STEME exhibits a significant lower performance than MEME, which can be caused by its nature design and implementation although its algorithm has similar properties to MEME [34]. In Fig. 3, the top three performers are Weeder, YMF, and Oligodyad-analysis. They outperform other tools while AlignACE, MITRA, and GLAM are the bottom three performers with GLAM is at the lowest rank. All top three performers in this figure are consensus-based methods. AlignACE and GLAM are profile-based methods. Although MITRA is a consensus-based method, it falls into the list of three bottom performers. This can be explained by the nature design and implementation of the tool. The profile-based methods are faster than consensus-based methods but they have lower accuracy than consensus-based methods because they tend to be trapped in a local optimum [37]. The observations for Figs. 2 and 3 confirm this fact except for MITRA.

Regardless of the poor performance, MOTIFSIM always reports the majority vote motif at the highest rank of similarity score. When we observe the performances of various tools on several sequence datasets, it shows that MOTIFSIM is more reliable for identifying the motifs that are more trustworthy than those reported by the poor performance tools. This is crucial particularly for the de novo motif finders because they do not use the reference database for verifying the found motifs. Thus, it may not be reliable for obtaining the results from individual de novo motif finders. The observation also indicates that using multiple tools for finding motifs and combining with MOTIFSIM for attaining the common significant motifs, it improved the results for DNA motif detection. This improvement is suitable for the general motif detection. If the motif discovery involves finding a specific type of motif by using a special tool, then using different types of motif finders may not be useful and MOTIFSIM is not recommended. On the other hand, because MOTIFSIM is specialized for motif similarity detection, the tool is useful for obtaining the common significant motifs from the results generated by several motif finders of the same type or by various motif finders of different types for the general motif detection. Besides, individual motif finders can be specialized for targeting different types of motifs. Hence, the users should select the most suitable method for their research for obtaining the best possible result.

## Conclusions

We compared the pair-wise comparison technique of MOTIFSIM with USW, AKL, ALLR, PCC, and CS for measuring similarity between DNA motifs. The comparison results show that MOTIFSIM achieves better performance than five methods above. We also compared MOTIFSIM with Matrix-clustering tool for clustering the motifs. The classification results on four taxonomic datasets demonstrate MOTIFSIM attains similar or better results than Matrix-clustering. Furthermore, we evaluated the performances of nineteen motif finders and the reliability of MOTIFSIM for identifying the common significant motifs. The comparison results reveal that some motif finders achieve better performance than other tools. Some failed to identify the known motif. However, when the motif detection does not require a special tool for finding a specific type of motif then using multiple tools for finding motifs and combining with MOTIFSIM for attaining the common significant motifs, it improved the results for DNA motif detection. Since individual motif finders can be specialized for different types of motifs, it is advisable to select the most suitable method for a particular type of research in order to achieve the best possible result.

## Additional file

Additional file 1: Supplementary Materials. (DOC 1779 kb)
