# Supplementary material for: Performance evaluation for MOTIFSIM
Source: Biol Proced Online. 2018 Dec 18;20:23. doi: 10.1186/s12575-018-0088-3 (PMC6299673; doi:10.1186/s12575-018-0088-3)
Supplement: Supplementary file 1 — Supplementary Materials. (DOC 1779 kb) [file 12575_2018_88_MOESM1_ESM.doc]

### Performance Evaluation for MOTIFSIM

Ngoc Tam L. Tran and Chun-Hsi Huang

### SUPPLEMENTARY MATERIALS

**MOTIFSIM Algorithm**

The algorithm has six steps as follows [1].

***1. Combine motifs from multiple datasets with different formats into one list M.***

Motifs from *n* input datasets in different formats are converted to (PSPMs) and combined into one list *M*. Their reverse complements are also calculated for comparisons.

***2. Perform pair-wise comparisons on the entire list M.***

This step performs forward and backward comparisons on each pair of matrices including their reverse complements in *M*.

***3. Calculate similarity score between two motifs***

For each position in previous step, for each pair of matrices (*p_i_, p_i+_*_1_) in a pair-wise comparison:

- 1. Calculate the absolute value of the difference between each pair of corresponding matrix elements
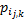
 and
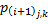
 in the overlapping window between two matrices *p_i_* and *p_(i+1)_* . The difference matrix is
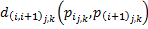
 .
  2. Calculate the average of the differences for each overlapping window between two matrices. The complement of this value is the similarity *s*(*_i,i_*_+1)_ between two matrices at that overlapping window. *s*(*_i,i_*_+1)_ is calculated as follows.


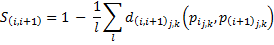


where *l* is the number of elements in the overlapping window. Higher value of *s*(*_i,i_*_+1)_ indicates more similarity between two matrices at that overlapping window.

- 1. Apply similarity filter on *s*(*_i,i_*_+1)_ by using the similarity cutoff *t* selected by the user. All *s*(*_i,i_*_+1)_ values falling below this threshold are filtered out.
  2. Calculate the distance *Ds*(*_i,i_*_+1)_ between *s*(*_i,i_*_+1)_ and the maximum of (*s*(*_i,i_*_+1)_, *s*(*_i,i_*_+2)_, *s*(*_i,i_*_+3)_, ..., *s*(*_i,m_*)). Smaller *Ds*(*_i,i_*_+1)_ shows higher similarity between two matrices.
  3. Calculate the distance *Do*(*_i,i_*_+1)_ between overlap window *o*(*_i,i_*_+1)_ and the maximum overlapping window of (*o*(*_i,i_*_+1)_, *o*(*_i,i_*_+2)_, *o*(*_i,i_*_+3)_, ..., *o*(*_i,m_*)). Smaller *Do*(*_i,i_*_+1)_ shows longer overlapping between two matrices.
  4. The similarity score *Sim*(*_i,i_*_+1)_ between two matrices is the average of *Ds*(*_i,i_*_+1)_ and *Do*(*_i,i_*_+1)_. Smaller *Sim*(*_i,i_*_+1)_ indicates higher similarity between two matrices.

Steps (iv-vi) above balance out the scores *s*(*_i,i_*_+1)_ for long overlapping windows and short overlapping windows between two matrices by taking the average of *Ds*(*_i,i_*_+1)_ and *Do*(*_i,i_*_+1)_ to derive the similarity score *Sim*(*_i,i_*_+1)_.

***4. Identify and report top k global significant motifs (k best matches) based on similarity score Sim*(*_i,i_*_+1_*).***

The global significant motifs are those reported by multiple tools. They are identified by comparing motifs in a dataset with motifs in other datasets. To select the top *k* global significant motifs, the similarity scores *Sim*(*_i,i_*_+1)_ between every pair of motifs in *M* are sorted in ascending order. The top *k* motifs with their *k* best matches in ascending order are selected from the sorted list.

***5. Identify and report k global and local significant motifs (k global and local best matches) based on similarity score Sim*(*_i,i_*_+1)_*.***

The global and local significant motifs are identified by comparing motifs locally in the same dataset as well as with other motifs in other datasets. The sorted list above is also used for selecting top *k* global and local significant motifs and their *k* best matches in ascending order.

***6. Identify and report k best matches for each motif in M using similarity score Sim*(*_i,i_*_+1_*).***

The best matches for each motif are identified by comparing each motif against other motifs in *M*. The motifs are reported in the order they are entered and combined in *M*. The top *k* best matches in ascending order for each motif are selected from the sorted list.

**Statistics used in Evaluation**

Tompa *et al.* measured the correctness of a tool *T* on a dataset *D* at both *nucleotide level* and at *site level*. At the nucleotide level, true positives (*nTP*), false negatives (*nFN*), false positive (*nFP*), and true negative (*nTN*) are defined as follows [2].

- *nTP*: the number of nucleotide positions in both known sites and predicted sites.
- *nFN*: the number of nucleotide positions in known sites but not in predicted sites.
- *nFP*: the number of nucleotide positions not in known sites but in predicted sites.
- *nTN*: the number of nucleotide positions in neither known sites nor predicted sites.

At the site level, a predicted site is considered overlapping a known site if they overlap by at least one-quarter the length of the known site. Similarly, true positives (*sTP*), false negatives (*sFN*), and false positives (*sFP*) are also defined as follows [2].

- *sTP*: the number of known sites overlapped by predicted sites.
- *sFN*: the number of known sites not overlapped by predicted sites.
- *sFP*: the number of predicted sites not overlapped by known sites.

Further, at nucleotide level (*x* = *n*) or site level (*x* = *s*), six statistics used in this validation are defined as follows [2].

- *Sensitivity*:
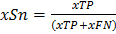


*xSn* gives the portion of known sites that are predicted.

- *Positive Predictive Value*:
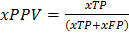


*xPPV* gives the portion of predicted sites that are known.

- *Specificity*:
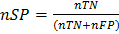


*nSP* gives the portion of nucleotides that are known neither in known sites nor in predicted sites.

- *Correlation Coefficient*:


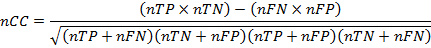


*nCC* is the Pearson product-moment coefficient of correlation. It measures the correlation between two sets of positions, which are the known nucleotide positions and the predicted nucleotide positions. *nCC* has the value ranging from -1 for perfect no correlation to +1 for perfect correlation. If the predicted motifs exactly match with the known binding sites, then *nCC* would be +1. Otherwise, if each nucleotide position was predicted to be in the motif randomly and independently, then the expected value of *nCC* would be 0 for no correlation [2].

**Tables**

**Table S1. Characteristics of 13 older motif finders used by Tompa *et al*. [2] and 6 newer motif finders**. The older tools are in *italic* face. The function, approach, and type of method for each tool are listed.

| **Tool** | **Function** | **Approach** | **Type** | **Year** | **Ref.** |
| --- | --- | --- | --- | --- | --- |
| *AlignACE* | Discover DNA regulatory motifs within unaligned non-coding sequences clustered by whole-genome mRNA quantitation. | Use Gibbs sampling method. | Profile-based method | 1998 | [2] |
| *ANN-Spec* | Discover DNA binding site motifs. | Use an Artificial Neural Network and a Gibbs sampling method. | Profile-based method | 2000 | [2] |
| ChIPMunk | Fast heuristic motif finder developed for analyzing high-throughput sequencing data. | Implemented an iterative approach that combines the greedy optimization with bootstrapping. | Profile-based method | 2010 | [3] |
| *Consensus* | Discover motifs in DNA and protein sequences. | Use multiple sequence alignment approach with maximum information content. | Profile-based method | 1999 | [2] |
| DMINDA | An integrated Web server with multiple functions for (1) *de novo* DNA motif finder, (2) scanning motif instances of a query motif, (3) motif comparison and clustering of identified motifs, and (4) co-occurrence analyses of query motifs in given promoter sequences. | Implemented BOBRO algorithm for prediction of cis-regulatory motifs in a given set of promoter sequences. The algorithm is based on finding motifs through finding cliques in a graph. | Graph-based method | 2014 | [4] |
| *GLAM* | Discover DNA motifs. | Use gapless local alignment of multiple sequences based on several enhancements to the Gibbs sampling alignment method. | Profile-based method | 2004 | [2] |
| *Improbizer* | Discover motifs in DNA or RNA sequences. | Use a variation of the expectation maximization algorithm. | Profile-based method | 2004 | [2] |
| *MEME (older version)* | Target un-gapped motifs in unaligned DNA or protein sequences. | Implemented Multiple Expectation Maximization for Motif Elicitation. | Profile-based method | Unspecified | [2] |
| MEME (v. 4.11.4) | Target un-gapped motifs in unaligned DNA or protein sequences. | Implemented Multiple Expectation Maximization for Motif Elicitation. | Profile-based method | 2017 | [5] |
| *MITRA* | Discover DNA motifs. | Implemented a mismatch tree algorithm (MITRA) to discover composite patterns. | Consensus-based method | 2002 | [2] |
| *MotifSampler* | Discover DNA motifs. | Extended Gibbs sampling algorithm for motif finding with a higher-order background model. | Profile-based method | 2001 | [2] |
| *Oligodyad-analysis* | Discover DNA motifs. | Implemented the dyad analysis method, which is based on the observation of regulatory sites consisting of a pair of highly conserved trinucleotides. The method counts the number of occurrences of each possible spaced pair of trinucleotides and assess its statistical significance. | Word-based method | 2000 | [2] |
| peak-motifs | Predict motifs from ChIP-Seq data. | Implemented Oligo-analysis, Position-analysis, and Local-word-analysis. | Word-based method | 2011 | [6] |
| *QuickScore* | Discover DNA motifs. | Based on an exhaustive search algorithm and estimates the probabilities of frequent words. | Consensus-based method | 2004 | [2] |
| *SeSiMCMC* | Discover DNA motifs. | Use a modification of the Gibbs sampling algorithm to find structured motifs and non-structured motifs in a set of unaligned DNA sequences. | Profile-based method | 2005 | [2] |
| STEME | Discover motifs in large datasets. | Implemented the Expectation Maximization (EM) approximation and extended it to fully-fledged motif finder with similar properties to MEME. | Profile-based method | 2014 | [7] |
| *Weeder* | Target DNA motifs. | Implemented a suffix tree based exhaustive enumeration and extended it for searching longer patterns. | Consensus-based method | Unspecified | [2] |
| XXmotif | General-purpose method designed for finding enriched motifs in nucleotide sequences. | Use a combination of the pattern-based enumerative approach and the iterative PWM refinement. | Profile-based method  Consensus-based method | 2012 | [8] |
| *YMF* | Discover motifs in DNA or RNA sequences. | Based on enumerative method and search for motifs with greatest z-scores. | Consensus-based method | 2003 | [2] |

**Table S2. Motif collection used in the first phase of the assessment**. The first one hundred and twelve are predicted motifs [2]. The rests are motifs from TRANSFAC database [9]. The origin indicates if a motif was generated by a tool or it came from the TRANSFAC database. The sequence dataset and the species also indicate where the motif came from.

| **No**. | **Motif** | **Predicted Motif** | **Origin** | **Sequence Dataset** | **Species** |
| --- | --- | --- | --- | --- | --- |
| 1 | AlignACE_hm08m | x | AlignACE | hm08m | Homo sapiens |
| 2 | ANN-Spec_hm08m | x | ANN-Spec |
| 3 | GLAM_hm08m | x | GLAM |
| 4 | Improbizer_hm08m | x | Improbizer |
| 5 | MEME_hm08m | x | MEME (older version) |
| 6 | MITRA_hm08m | x | MITRA |
| 7 | MotifSampler_hm08m | x | MotifSampler |
| 8 | oligodyad-analysis_hm08m | x | oligodyad-analysis |
| 9 | QuickScore_hm08m | x | QuickScore |
| 10 | SeSiMCMC_hm08m | x | SeSiMCMC |
| 11 | Weeder_hm08m | x | Weeder |
| 12 | YMF_hm08m | x | YMF |
| 13 | ANN-Spec_hm22m | x | ANN-Spec | hm22m | Homo sapiens |
| 14 | GLAM_hm22m | x | GLAM |
| 15 | Improbizer_hm22m | x | Improbizer |
| 16 | MEME_hm22m | x | MEME (older version) |
| 17 | MITRA_hm22m | x | MITRA |
| 18 | MotifSampler_hm22m | x | MotifSampler |
| 19 | oligodyad-analysis_hm22m | x | oligodyad-analysis |
| 20 | SeSiMCMC_hm22m | x | SeSiMCMC |
| 21 | Weeder_hm22m | x | Weeder |
| 22 | YMF_hm22m | x | YMF |
| 23 | AlignACE_mus04m | x | AlignACE | mus04m | Mus musculus |
| 24 | ANN-Spec_mus04m | x | ANN-Spec |
| 25 | Consensus_mus04m | x | Consensus |
| 26 | GLAM_mus04m | x | GLAM |
| 27 | Improbizer_mus04m | x | Improbizer |
| 28 | MEME_mus04m | x | MEME (older version) |
| 29 | MITRA_mus04m | x | MITRA |
| 30 | MotifSampler_mus04m | x | MotifSampler |
| 31 | oligodyad-analysis_mus04m | x | oligodyad-analysis |
| 32 | SeSiMCMC_mus04m | x | SeSiMCMC |
| 33 | Weeder_mus04m | x | Weeder |
| 34 | YMF_mus04m | x | YMF |
| 35 | AlignACE_mus06g | x | AlignACE | mus06g | Mus musculus |
| 36 | ANN-Spec_mus06g | x | ANN-Spec |
| 37 | Consensus_mus06g | x | Consensus |
| 38 | GLAM_mus06g | x | GLAM |
| 39 | Improbizer_mus06g | x | Improbizer |
| 40 | MEME_mus06g | x | MEME (older version) |
| 41 | MITRA_mus06g | x | MITRA |
| 42 | MotifSampler_mus06g | x | MotifSampler |
| 43 | oligodyad-analysis_mus06g | x | oligodyad-analysis |
| 44 | QuickScore_mus06g | x | QuickScore |
| 45 | SeSiMCMC_mus06g | x | SeSiMCMC |
| 46 | Weeder_mus06g | x | Weeder |
| 47 | YMF_mus06g | x | YMF |
| 48 | AlignACE_mus10g | x | AlignACE | mus10g | Mus musculus |
| 49 | ANN-Spec_mus10g | x | ANN-Spec |
| 50 | Consensus_mus10g | x | Consensus |
| 51 | GLAM_mus10g | x | GLAM |
| 52 | Improbizer_mus10g | x | Improbizer |
| 53 | MITRA_mus10g | x | MITRA |
| 54 | oligodyad-analysis_mus10g | x | oligodyad-analysis |
| 55 | QuickScore_mus10g | x | QuickScore |
| 56 | SeSiMCMC_mus10g | x | SeSiMCMC |
| 57 | Weeder_mus10g | x | Weeder |
| 58 | YMF_mus10g | x | YMF |
| 59 | AlignACE_mus11m | x | AlignACE | mus11m | Mus musculus |
| 60 | ANN-Spec_mus11m | x | ANN-Spec |
| 61 | Consensus_mus11m | x | Consensus |
| 62 | GLAM_mus11m | x | GLAM |
| 63 | Improbizer_mus11m | x | Improbizer |
| 64 | MEME_mus11m | x | MEME (older version) |
| 65 | MITRA_mus11m | x | MITRA |
| 66 | MotifSampler_mus11m | x | MotifSampler |
| 67 | SeSiMCMC_mus11m | x | SeSiMCMC |
| 68 | Weeder_mus11m | x | Weeder |
| 69 | YMF_mus11m | x | YMF |
| 70 | ANN-Spec_yst02g | x | ANN-Spec | yst02g | Saccharomyces cerevisiae |
| 71 | GLAM_yst02g | x | GLAM |
| 72 | Improbizer_yst02g | x | Improbizer |
| 73 | MEME_yst02g | x | MEME (older version) |
| 74 | MITRA_yst02g | x | MITRA |
| 75 | MotifSampler_yst02g | x | MotifSampler |
| 76 | oligodyad-analysis_yst02g | x | oligodyad-analysis |
| 77 | QuickScore_yst02g | x | QuickScore |
| 78 | SeSiMCMC_yst02g | x | SeSiMCMC |
| 79 | Weeder_yst02g | x | Weeder |
| 80 | YMF_yst02g | x | YMF |
| 81 | AlignACE_yst03m | x | AlignACE | yst03m | Saccharomyces cerevisiae |
| 82 | ANN-Spec_yst03m | x | ANN-Spec |
| 83 | Consensus_yst03m | x | Consensus |
| 84 | GLAM_yst03m | x | GLAM |
| 85 | Improbizer_yst03m | x | Improbizer |
| 86 | MEME_yst03m | x | MEME (older version) |
| 87 | MITRA_yst03m | x | MITRA |
| 88 | MotifSampler_yst03m | x | MotifSampler |
| 89 | oligodyad-analysis_yst03m | x | oligodyad-analysis |
| 90 | QuickScore_yst03m | x | QuickScore |
| 91 | SeSiMCMC_yst03m | x | SeSiMCMC |
| 92 | Weeder_yst03m | x | Weeder |
| 93 | YMF_yst03m | x | YMF |
| 94 | ANN-Spec_yst06g | x | ANN-Spec | yst06g | Saccharomyces cerevisiae |
| 95 | GLAM_yst06g | x | GLAM |
| 96 | Improbizer_yst06g | x | Improbizer |
| 97 | MEME_yst06g | x | MEME (older version) |
| 98 | MITRA_yst06g | x | MITRA |
| 99 | MotifSampler_yst06g | x | MotifSampler |
| 100 | oligodyad-analysis_yst06g | x | oligodyad-analysis |
| 101 | QuickScore_yst06g | x | QuickScore |
| 102 | SeSiMCMC_yst06g | x | SeSiMCMC |
| 103 | Weeder_yst06g | x | Weeder |
| 104 | YMF_yst06g | x | YMF |
| 105 | ChIPMunk_hm17g | x | ChIPMunk | hm17g | Homo sapiens |
| 106 | DMINDA_hm17g | x | DMINDA |
| 107 | MEME_hm17g | x | MEME |
| 108 | peak-motifs_hm17g | x | peak-motifs |
| 109 | XXMotif_hm17g | x | XXMotif |
| 110 | ChIPMunk_yst09g | x | ChIPMunk | yst09g | Saccharomyces cerevisiae |
| 111 | DMINDA_yst09g | x | DMINDA |
| 112 | peak-motifs_yst09g | x | peak-motifs |
| 113 | Abdominal-B |  | TRANSFAC database |  | Drosophila melanogaster |
| 114 | Adf-1 |  |
| 115 | BR-C-Z1 |  |
| 116 | Dfd |  |
| 117 | E74A |  |
| 118 | Ftz |  |
| 119 | Ovo |  |
| 120 | AP-4 |  | TRANSFAC database |  | Homo sapiens |
| 121 | Arnt |  |
| 122 | ATF6 |  |
| 123 | CDP-CR1 |  |
| 124 | c-Myc:Max |  |
| 125 | CREB |  |
| 126 | E47 |  |
| 127 | Egr-3 |  |
| 128 | Elk-1 |  |
| 129 | FOXO1 |  |
| 130 | Freac-2 |  |
| 131 | GATA-X |  |
| 132 | MEIS1A:HOXA9 |  |
| 133 | NF-kappaB |  |
| 134 | Nkx2-2 |  |
| 135 | RORalpha1 |  |
| 136 | STAT1 |  |
| 137 | STAT4 |  |
| 138 | STAT5A_homodimer |  |
| 139 | AP-2alpha |  | TRANSFAC database |  | Mus musculus |
| 140 | Arnt |  |
| 141 | c-Myc:Max |  |
| 142 | c-Rel |  |
| 143 | FOXO1 |  |
| 144 | HFH-3 |  |
| 145 | HSF1 |  |
| 146 | Ik-1 |  |
| 147 | IRF-1 |  |
| 148 | MEIS1A:HOXA9 |  |
| 149 | Nkx2-2 |  |
| 150 | Pax-6 |  |
| 151 | STAT1 |  |
| 152 | STAT4 |  |
| 153 | STAT5A_homodimer |  |
| 154 | ADR1 |  | TRANSFAC database |  | Saccharomyces cerevisiae |
| 155 | GAL4 |  |
| 156 | HAP2-3-4 |  |
| 157 | HSF |  |
| 158 | MATa1 |  |

**Table S3. Motif dataset for Fungi group**. The motifs are grouped by family that they belong in the Jaspar database [10]. The dataset contains 78 motifs that belong to 11 families.

| **No.** | **Motif ID** | **Motif Name** | **Family** |
| --- | --- | --- | --- |
| 1 | MA0265.1 | ABF1 | BetaBetaAlpha-zinc finger Family |
| 2 | MA0277.1 | AZF1 |
| 3 | MA0333.1 | MET31 |
| 4 | MA0366.1 | RGM1 |
| 5 | MA0373.1 | RPN4 |
| 6 | MA0396.1 | STP3 |
| 7 | MA0397.1 | STP4 |
| 8 | MA0402.1 | SWI5 |
| 9 | MA0413.1 | USV1 |
| 10 | MA0298.1 | FZF1 | Factors with multiple dispersed zinc fingers |
| 11 | MA0364.1 | REI1 |
| 12 | MA0368.1 | RIM101 |
| 13 | MA0370.1 | RME1 |
| 14 | MA0378.1 | SFP1 |
| 15 | MA0394.1 | STP1 |
| 16 | MA0395.1 | STP2 |
| 17 | MA0440.1 | ZAP1 |
| 18 | MA0295.1 | FHL1 | Forkhead box (FOX) factors |
| 19 | MA0296.1 | FKH1 |
| 20 | MA0297.1 | FKH2 |
| 21 | MA0317.1 | HCM1 |
| 22 | MA0929.1 | NCU00019 |
| 23 | MA0276.1 | ASH1 | GATA-type zinc fingers |
| 24 | MA0289.1 | DAL80 |
| 25 | MA0293.1 | ECM23 |
| 26 | MA0300.1 | GAT1 |
| 27 | MA0301.1 | GAT3 |
| 28 | MA0302.1 | GAT4 |
| 29 | MA0307.1 | GLN3 |
| 30 | MA0309.1 | GZF3 |
| 31 | MA0389.1 | SRD1 |
| 32 | MA0281.1 | CBF1 | Helix-Loop-Helix Family |
| 33 | MA0321.1 | INO2 |
| 34 | MA0322.1 | INO4 |
| 35 | MA0357.1 | PHO4 |
| 36 | MA0409.1 | TYE7 |
| 37 | MA0266.1 | ABF2 | High Mobility Group (Box) Family |
| 38 | MA0344.1 | NHP10 |
| 39 | MA0345.1 | NHP6A |
| 40 | MA0346.1 | NHP6B |
| 41 | MA0371.1 | ROX1 |
| 42 | MA0387.1 | SPT2 |
| 43 | MA0356.1 | PHO2 | Homeodomain Family |
| 44 | MA0393.1 | STE12 |
| 45 | MA0406.1 | TEC1 |
| 46 | MA0426.1 | YHP1 |
| 47 | MA0433.1 | YOX1 |
| 48 | MA0319.1 | HSF1 | HSF factors |
| 49 | MA0336.1 | MGA1 |
| 50 | MA0377.1 | SFL1 |
| 51 | MA0381.1 | SKN7 |
| 52 | MA0274.1 | ARR1 | Leucine Zipper Family |
| 53 | MA0279.1 | CAD1 |
| 54 | MA0284.1 | CIN5 |
| 55 | MA0286.1 | CST6 |
| 56 | MA0303.1 | GCN4 |
| 57 | MA0310.1 | HAC1 |
| 58 | MA0332.1 | MET28 |
| 59 | MA0335.1 | MET4 |
| 60 | MA0349.1 | OPI1 |
| 61 | MA0382.1 | SKO1 |
| 62 | MA0415.1 | YAP1 |
| 63 | MA0416.1 | YAP3 |
| 64 | MA0417.1 | YAP5 |
| 65 | MA0418.1 | YAP6 |
| 66 | MA0419.1 | YAP7 |
| 67 | MA0278.1 | BAS1 | Myb/SANT domain factors |
| 68 | MA0351.1 | DOT6 |
| 69 | MA0421.1 | NSI1 |
| 70 | MA0359.1 | RAP1 |
| 71 | MA0363.1 | REB1 |
| 72 | MA0384.1 | SNT2 |
| 73 | MA0403.1 | TBF1 |
| 74 | MA0350.1 | TOD6 |
| 75 | MA0288.1 | CUP9 | TALE-type homeo domain factors |
| 76 | MA0318.1 | HMRA2 |
| 77 | MA0328.2 | MATALPHA2 |
| 78 | MA0408.1 | TOS8 |

**Table S4. Motif dataset for Insects group**. The motifs are grouped by family that they belong in the Jaspar database [10]. The dataset contains 42 motifs that belong to 7 different families.

| **No.** | **Motif ID** | **Motif Name** | **Family** |
| --- | --- | --- | --- |
| 1 | MA0010.1 | br | BetaBetaAlpha-zinc finger Family |
| 2 | MA0531.1 | CTCF |
| 3 | MA0049.1 | hb |
| 4 | MA0126.1 | ovo |
| 5 | MA0244.1 | slbo |
| 6 | MA0086.1 | sna |
| 7 | MA0205.1 | Trl |
| 8 | MA0015.1 | Cf2 | Factors with multiple dispersed zinc fingers |
| 9 | MA0533.1 | su(Hw) |
| 10 | MA0446.1 | fkh | Forkhead box (FOX) factors |
| 11 | MA0458.1 | slp1 |
| 12 | MA0449.1 | h | Helix-Loop-Helix Family |
| 13 | MA0249.1 | twi |
| 14 | MA0210.1 | ara | Homeodomain Family |
| 15 | MA0211.1 | bap |
| 16 | MA0212.1 | bcd |
| 17 | MA0214.1 | bsh |
| 18 | MA0215.1 | btn |
| 19 | MA0217.1 | caup |
| 20 | MA0219.1 | ems |
| 21 | MA0220.1 | en |
| 22 | MA0221.1 | eve |
| 23 | MA0222.1 | exd |
| 24 | MA0224.1 | exex |
| 25 | MA0225.1 | ftz |
| 26 | MA0226.1 | hbn |
| 27 | MA0227.1 | hth |
| 28 | MA0228.1 | ind |
| 29 | MA0229.1 | inv |
| 30 | MA0230.1 | lab |
| 31 | MA0231.1 | lbe |
| 32 | MA0232.1 | lbl |
| 33 | MA0233.1 | mirr |
| 34 | MA0245.1 | slou |
| 35 | MA0452.2 | Kr | More than 3 adjacent zinc finger factors |
| 36 | MA0454.1 | odd |
| 37 | MA0456.1 | opa |
| 38 | MA0011.1 | br(var.2) | Other factors with up to three adjacent zinc fingers |
| 39 | MA0012.1 | br(var.3) |
| 40 | MA0013.1 | br(var.4) |
| 41 | MA0443.1 | btd |
| 42 | MA0460.1 | ttk |

**Table S5. Motif dataset for Plants group**. The motifs are grouped by family that they belong in the Jaspar database [10]. The dataset contains 65 motifs that belong to 6 different families.

| **No.** | **Motif ID** | **Motif Name** | **Family** |
| --- | --- | --- | --- |
| 1 | MA0973.1 | CDF2 | Dof-type |
| 2 | MA0974.1 | CDF3 |
| 3 | MA0981.1 | DOF1.8 |
| 4 | MA0020.1 | Dof2 |
| 5 | MA0982.1 | DOF2.4 |
| 6 | MA0977.1 | DOF2.5 |
| 7 | MA0021.1 | Dof3 |
| 8 | MA1071.1 | DOF5.3 |
| 9 | MA0983.1 | DOF5.6 |
| 10 | MA0984.1 | DOF5.7 |
| 11 | MA0053.1 | MNB1A |
| 12 | MA0064.1 | PBF |
| 13 | MA0987.1 | PHYPADRAFT_140773 |
| 14 | MA0989.1 | PHYPADRAFT_153324 |
| 15 | MA1022.1 | PHYPADRAFT_38837 |
| 16 | MA1013.1 | GATA10 | GATA-type zinc fingers |
| 17 | MA1014.1 | GATA11 |
| 18 | MA1015.1 | GATA12 |
| 19 | MA1016.1 | GATA15 |
| 20 | MA1017.1 | GATA8 |
| 21 | MA1018.1 | GATA9 |
| 22 | MA0950.1 | ATHB-12 | HD-ZIP factors |
| 23 | MA1026.1 | ATHB-15 |
| 24 | MA0951.1 | ATHB-16 |
| 25 | MA0952.1 | ATHB-51 |
| 26 | MA0953.1 | ATHB-6 |
| 27 | MA0954.1 | ATHB-7 |
| 28 | MA0990.1 | EDT1 |
| 29 | MA1024.1 | HAT1 |
| 30 | MA0096.1 | bZIP910 | Leucine Zipper Family |
| 31 | MA0097.1 | bZIP911 |
| 32 | MA0128.1 | EmBP-1 |
| 33 | MA0127.1 | PEND |
| 34 | MA0129.1 | TGA1A |
| 35 | MA0945.1 | ARR1 | Myb/SANT domain factors |
| 36 | MA0121.1 | ARR10 |
| 37 | MA0946.1 | ARR11 |
| 38 | MA0947.1 | ARR14 |
| 39 | MA0948.1 | ARR18 |
| 40 | MA0949.1 | ARR2 |
| 41 | MA0972.1 | CCA1 |
| 42 | MA0579.1 | CDC5 |
| 43 | MA0575.1 | F3A4.140 |
| 44 | MA0034.1 | Gam1 |
| 45 | MA1020.1 | GT-1 |
| 46 | MA1027.1 | KAN1 |
| 47 | MA0054.1 | myb.Ph3 |
| 48 | MA0574.1 | MYB15 |
| 49 | MA0576.1 | RAX3 |
| 50 | MA1098.1 | ARALYDRAFT_484486 | TCP domain |
| 51 | MA1097.1 | ARALYDRAFT_493022 |
| 52 | MA1095.1 | ARALYDRAFT_495258 |
| 53 | MA1096.1 | ARALYDRAFT_496250 |
| 54 | MA1054.1 | ARALYDRAFT_897773 |
| 55 | MA1019.1 | Glyma19g26560.1 |
| 56 | MA1031.1 | OJ1581_H09.2 |
| 57 | MA1050.1 | OsI_08196 |
| 58 | MA1062.1 | TCP15 |
| 59 | MA0587.1 | TCP16 |
| 60 | MA1063.1 | TCP19 |
| 61 | MA1064.1 | TCP2 |
| 62 | MA1065.1 | TCP20 |
| 63 | MA1066.1 | TCP23 |
| 64 | MA1035.1 | TCP4 |
| 65 | MA1067.1 | TCP5 |

**Table S6. Motif dataset for Vertebrates group**. The motifs are grouped by family that they belong in the Jaspar database [10]. The dataset consists of 73 motifs that belong to 9 families.

| **No.** | **Motif ID** | **Motif Name** | **Family** |
| --- | --- | --- | --- |
| 1 | MA0003.3 | TFAP2A | AP-2 |
| 2 | MA0810.1 | TFAP2A(var.2) |
| 3 | MA0872.1 | TFAP2A(var.3) |
| 4 | MA0811.1 | TFAP2B |
| 5 | MA0812.1 | TFAP2B(var.2) |
| 6 | MA0813.1 | TFAP2B(var.3) |
| 7 | MA0524.2 | TFAP2C |
| 8 | MA0814.1 | TFAP2C(var.2) |
| 9 | MA0815.1 | TFAP2C(var.3) |
| 10 | MA0102.3 | CEBPA | C/EBP-related |
| 11 | MA0466.2 | CEBPB |
| 12 | MA0836.1 | CEBPD |
| 13 | MA0837.1 | CEBPE |
| 14 | MA0838.1 | CEBPG |
| 15 | MA0639.1 | DBP |
| 16 | MA0019.1 | Ddit3::Cebpa |
| 17 | MA0043.2 | HLF |
| 18 | MA0025.1 | NFIL3 |
| 19 | MA0604.1 | Atf1 | CREB-related factors |
| 20 | MA0018.2 | CREB1 |
| 21 | MA0638.1 | CREB3 |
| 22 | MA0839.1 | CREB3L1 |
| 23 | MA0608.1 | Creb3l2 |
| 24 | MA0840.1 | Creb5 |
| 25 | MA0609.1 | Crem |
| 26 | MA0024.3 | E2F1 | E2F-related factors |
| 27 | MA0864.1 | E2F2 |
| 28 | MA0469.2 | E2F3 |
| 29 | MA0470.1 | E2F4 |
| 30 | MA0471.1 | E2F6 |
| 31 | MA0758.1 | E2F7 |
| 32 | MA0865.1 | E2F8 |
| 33 | MA0739.1 | Hic1 | Factors with multiple dispersed zinc fingers |
| 34 | MA0738.1 | HIC2 |
| 35 | MA0131.2 | HINFP |
| 36 | MA0155.1 | INSM1 |
| 37 | MA0029.1 | Mecom |
| 38 | MA0138.2 | REST |
| 39 | MA0073.1 | RREB1 |
| 40 | MA0116.1 | Znf423 |
| 41 | MA0464.2 | BHLHE40 | Hairy-related factors |
| 42 | MA0636.1 | BHLHE41 |
| 43 | MA1099.1 | Hes1 |
| 44 | MA0616.1 | Hes2 |
| 45 | MA0821.1 | HES5 |
| 46 | MA0822.1 | HES7 |
| 47 | MA0823.1 | HEY1 |
| 48 | MA0649.1 | HEY2 |
| 49 | MA0914.1 | ISL2 | HD-LIM factors |
| 50 | MA0700.1 | LHX2 |
| 51 | MA0135.1 | Lhx3 |
| 52 | MA0704.1 | Lhx4 |
| 53 | MA0658.1 | LHX6 |
| 54 | MA0705.1 | Lhx8 |
| 55 | MA0701.1 | LHX9 |
| 56 | MA0702.1 | LMX1A |
| 57 | MA0703.1 | LMX1B |
| 58 | MA0007.3 | Ar | Steroid hormone receptors (NR3) |
| 59 | MA0112.3 | ESR1 |
| 60 | MA0258.2 | ESR2 |
| 61 | MA0592.2 | Esrra |
| 62 | MA0141.3 | ESRRB |
| 63 | MA0643.1 | Esrrg |
| 64 | MA0113.3 | NR3C1 |
| 65 | MA0727.1 | NR3C2 |
| 66 | MA0498.2 | MEIS1 | TALE-type homeo domain factors |
| 67 | MA0774.1 | MEIS2 |
| 68 | MA0775.1 | MEIS3 |
| 69 | MA0070.1 | PBX1 |
| 70 | MA0782.1 | PKNOX1 |
| 71 | MA0783.1 | PKNOX2 |
| 72 | MA0796.1 | TGIF1 |
| 73 | MA0797.1 | TGIF2 |

**Table S7. Motif collection was used in the third phase of the assessment**. Motif type indicates if a motif was generated by a newer tool or it came from Tompa *et al* [2]. The generated motifs came from 6 newer tools. The on-line motifs were collected from 13 older tools in Tompa *et al* [2].

| **No.** | **Motif** | **Total # of Motifs** | **Motif Type** | **Sequence Dataset** |
| --- | --- | --- | --- | --- |
| 1 | ChIPMunk_Motif1_hm01g | 6 | Generated motif | hm01g |
| 2 | DMINDA_Motif-32_3rd_ hm01g |
| 3 | MEME_Motif3_3rd_ hm01g |
| 4 | peak-motifs_Motif7_3rd_ hm01g |
| 5 | STEME_Motif1_1st_ hm01g |
| 6 | XXMotif_Motif1_1st_ hm01g |
| 7 | ChIPMunk_Motif1_ hm04g | 6 | Generated motif | hm04g |
| 8 | DMINDA_Motif-16_2nd_ hm04g |
| 9 | MEME_Motif1_1st_ hm04g |
| 10 | peak-motifs_Motif1_1st_ hm04g |
| 11 | STEME_Motif3_3rd_ hm04g |
| 12 | XXMotif_Motif3_3rd_ hm04g |
| 13 | ChIPMunk_Motif1_ hm15g | 6 | Generated motif | hm15g |
| 14 | DMINDA_Motif-19_1st_ hm15g |
| 15 | MEME_Motif1_1st_ hm15g |
| 16 | peak-motifs_Motif1_2nd_ hm15g |
| 17 | STEME_Motif3_3rd_ hm15g |
| 18 | XXMotif_Motif2_2nd_ hm15g |
| 19 | ChIPMunk_Motif1_1st_ hm17g | 5 | Generated motif | hm17g |
| 20 | DMINDA_Motif-16_1st_ hm17g |
| 21 | MEME_Motif1_1st_ hm17g |
| 22 | peak-motifs_Motif1_2nd_ hm17g |
| 23 | XXMotif_Motif1_1st_ hm17g |
| 24 | ChIPMunk_Motif1_1st_ hm19g | 3 | Generated motif | hm19g |
| 25 | DMINDA_Motif-29_2nd_ hm19g |
| 26 | peak-motifs_Motif1_2nd_ hm19g |
| 27 | ChIPMunk_Motif1_1st_ hm22g | 4 | Generated motif | hm22g |
| 28 | DMINDA_Motif-22_3rd_ hm22g |
| 29 | peak-motifs_Motif2_3rd _hm22g |
| 30 | STEME_Motif1_1st _hm22g |
| 31 | ChIPMunk_Motif1_1st_ yst09g | 3 | Generated motif | yst09g |
| 32 | DMINDA_Motif-8_1st_ yst09g |
| 33 | peak-motifs_Motif3_1st_ yst09g |
| 34 | AlignACE_hm08m | 12 | On-line motif | hm08m |
| 35 | ANN-Spec_hm08m |
| 36 | GLAM_hm08m |
| 37 | Improbizer_hm08m |
| 38 | MEME_hm08m |
| 39 | MITRA_hm08m |
| 40 | MotifSampler_hm08m |
| 41 | oligodyad-analysis_hm08m |
| 42 | QuickScore_hm08m |
| 43 | SeSiMCMC_hm08m |
| 44 | Weeder_hm08m |
| 45 | YMF_hm08m |
| 46 | ANN-Spec_hm22m | 10 | On-line motif | hm22m |
| 47 | GLAM_hm22m |
| 48 | Improbizer_hm22m |
| 49 | MEME_hm22m |
| 50 | MITRA_hm22m |
| 51 | MotifSampler_hm22m |
| 52 | oligodyad-analysis_hm22m |
| 53 | SeSiMCMC_hm22m |
| 54 | Weeder_hm22m |
| 55 | YMF_hm22m |
| 56 | AlignACE_mus04m | 12 | On-line motif | mus04m |
| 57 | ANN-Spec_mus04m |
| 58 | Consensus_mus04m |
| 59 | GLAM_mus04m |
| 60 | Improbizer_mus04m |
| 61 | MEME_mus04m |
| 62 | MITRA_mus04m |
| 63 | MotifSampler_mus04m |
| 64 | oligodyad-analysis_mus04m |
| 65 | SeSiMCMC_mus04m |
| 66 | Weeder_mus04m |
| 67 | YMF_mus04m |
| 68 | AlignACE_mus06g | 13 | On-line motif | mus06g |
| 69 | ANN-Spec_mus06g |
| 70 | Consensus_mus06g |
| 71 | GLAM_mus06g |
| 72 | Improbizer_mus06g |
| 73 | MEME_mus06g |
| 74 | MITRA_mus06g |
| 75 | MotifSampler_mus06g |
| 76 | oligodyad-analysis_mus06g |
| 77 | QuickScore_mus06g |
| 78 | SeSiMCMC_mus06g |
| 79 | Weeder_mus06g |
| 80 | YMF_mus06g |
| 81 | AlignACE_mus10g | 11 | On-line motif | mus10g |
| 82 | ANN-Spec_mus10g |
| 83 | Consensus_mus10g |
| 84 | GLAM_mus10g |
| 85 | Improbizer_mus10g |
| 86 | MITRA_mus10g |
| 87 | oligodyad-analysis_mus10g |
| 88 | QuickScore_mus10g |
| 89 | SeSiMCMC_mus10g |
| 90 | Weeder_mus10g |
| 91 | YMF_mus10g |
| 92 | AlignACE_mus11m | 11 | On-line motif | mus11m |
| 93 | ANN-Spec_mus11m |
| 94 | GLAM_mus11m |
| 95 | Improbizer_mus11m |
| 96 | MEME_mus11m |
| 97 | MITRA_mus11m |
| 98 | MotifSampler_mus11m |
| 99 | SeSiMCMC_mus11m |
| 100 | Consensus_mus11m |
| 101 | Weeder_mus11m |
| 102 | YMF_mus11m |
| 103 | ANN-Spec_yst02g | 11 | On-line motif | yst02g |
| 104 | GLAM_yst02g |
| 105 | Improbizer_yst02g |
| 106 | MEME_yst02g |
| 107 | MITRA_yst02g |
| 108 | MotifSampler_yst02g |
| 109 | oligodyad-analysis_yst02g |
| 110 | QuickScore_yst02g |
| 111 | SeSiMCMC_yst02g |
| 112 | Weeder_yst02g |
| 113 | YMF_yst02g |
| 114 | AlignACE_yst03m | 13 | On-line motif | yst03m |
| 115 | ANN-Spec_yst03m |
| 116 | Consensus_yst03m |
| 117 | GLAM_yst03m |
| 118 | Improbizer_yst03m |
| 119 | MEME_yst03m |
| 120 | MITRA_yst03m |
| 121 | MotifSampler_yst03m |
| 122 | oligodyad-analysis_yst03m |
| 123 | QuickScore_yst03m |
| 124 | SeSiMCMC_yst03m |
| 125 | Weeder_yst03m |
| 126 | YMF_yst03m |
| 127 | ANN-Spec_yst06g | 11 | On-line motif | yst06g |
| 128 | GLAM_yst06g |
| 129 | Improbizer_yst06g |
| 130 | MEME_yst06g |
| 131 | MITRA_yst06g |
| 132 | MotifSampler_yst06g |
| 133 | oligodyad-analysis_yst06g |
| 134 | QuickScore_yst06g |
| 135 | SeSiMCMC_yst06g |
| 136 | Weeder_yst06g |
| 137 | YMF_yst06g |

**Figures**

**Figure S1. Motif tree for the Fungi dataset in Table S3.** The tree was generated by Phylodendron for the result obtained by Matrix-clustering tool [11, 12]. The tool achieves 58% correct classification of 78 motifs into their families.


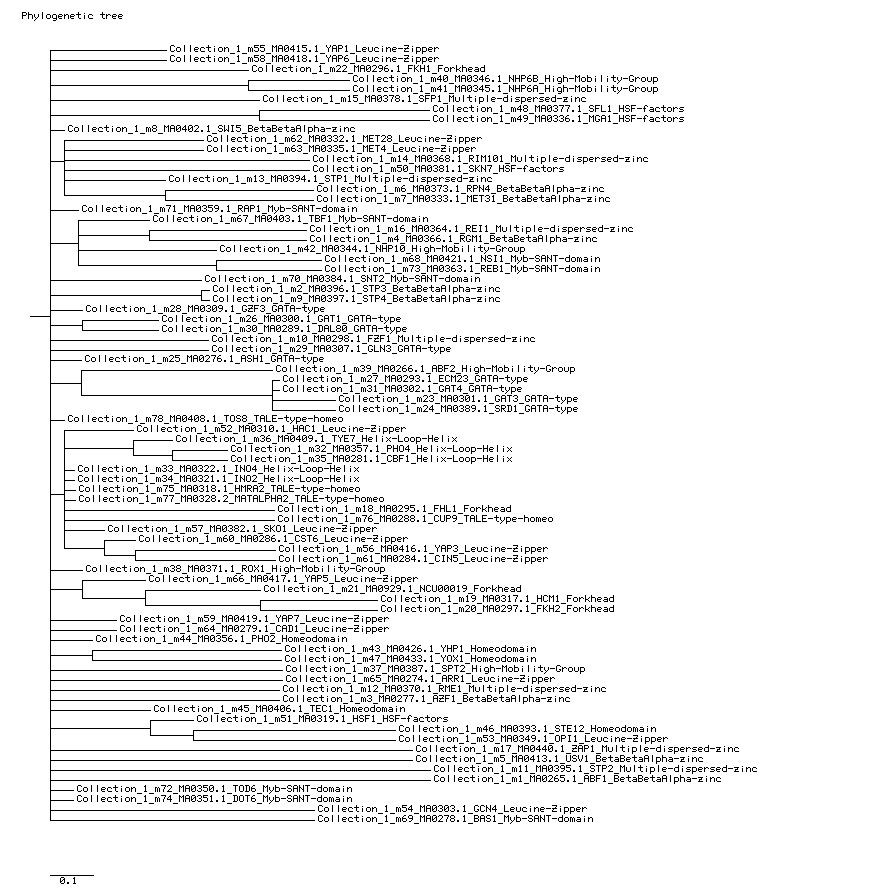


**Figure S2. Motif tree for the Fungi dataset in Table S3.** The tree was generated by MOTIFSIM tool [13]. The tool achieves 62% correct classification of 78 motifs into their families.


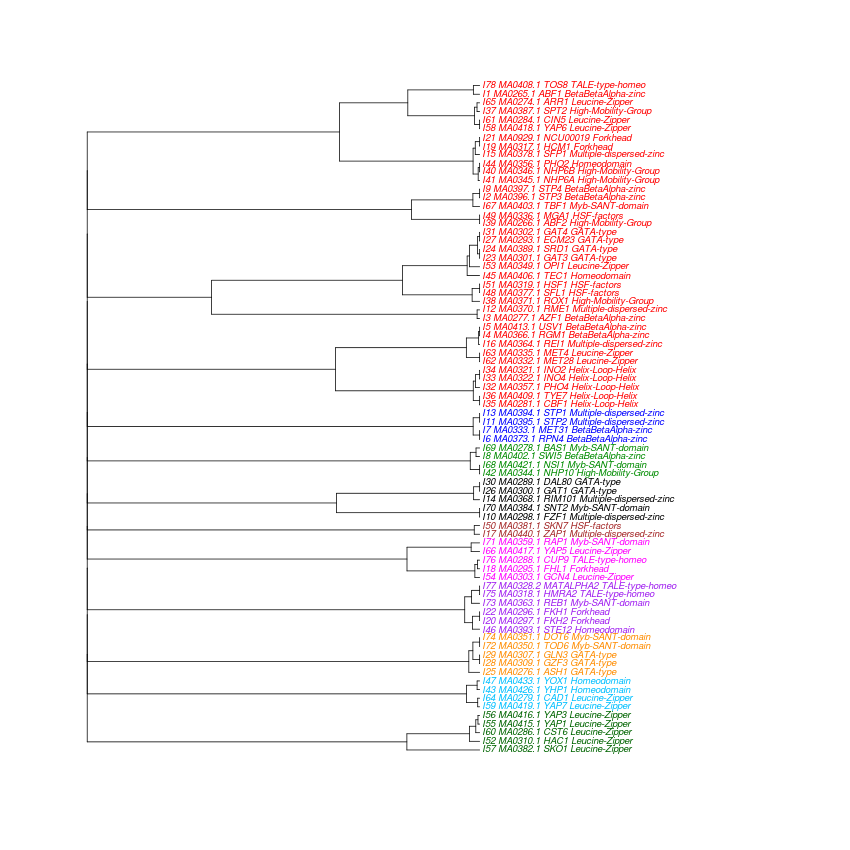


**Figure S3. Motif tree for the Insects dataset in Table S4.** The tree was generated by Phylodendron for the result obtained by Matrix-clustering tool [11, 12]. The tool achieves 55% correct classification of 42 motifs into their families.


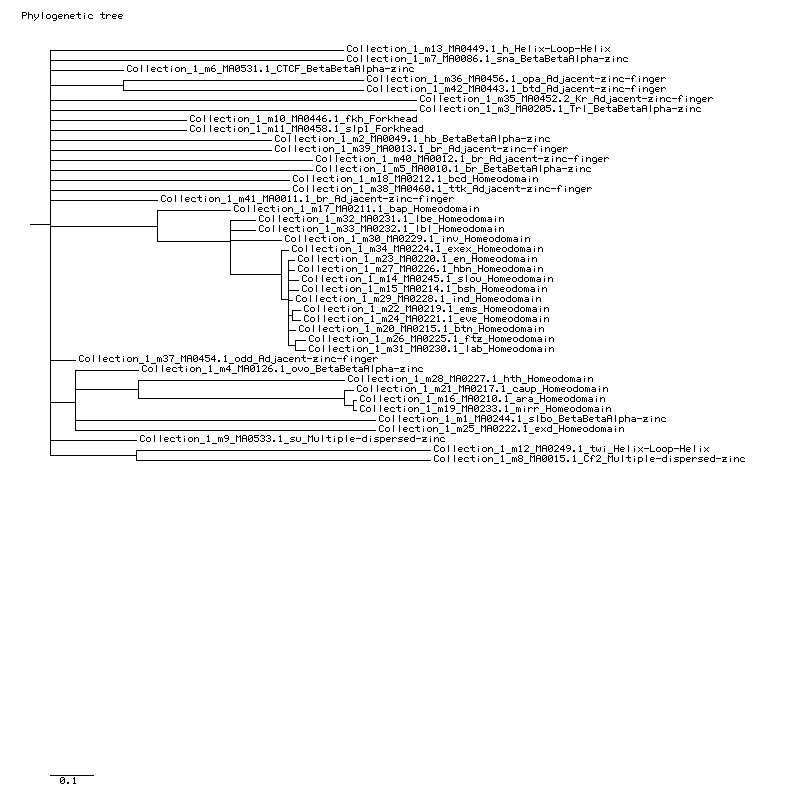


**Figure S4. Motif tree for the Insects dataset in Table S4.** The tree was generated by MOTIFSIM tool [13]. The tool achieves 57% correct classification of 42 motifs into their families.


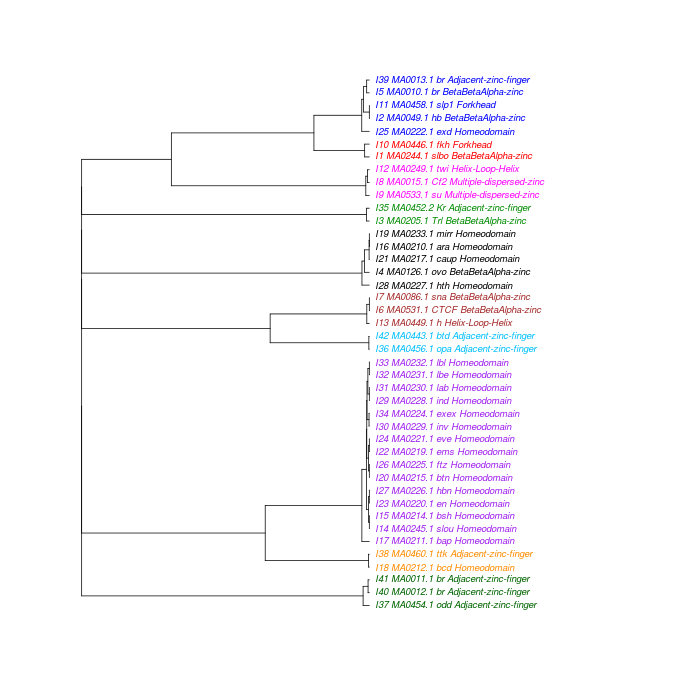


**Figure S5. Motif tree for the Plants dataset in Table S5.** The tree was generated by Phylodendron for the result obtained by Matrix-clustering tool [11, 12]. The tool achieves 97% correct classification of 65 motifs into their families.


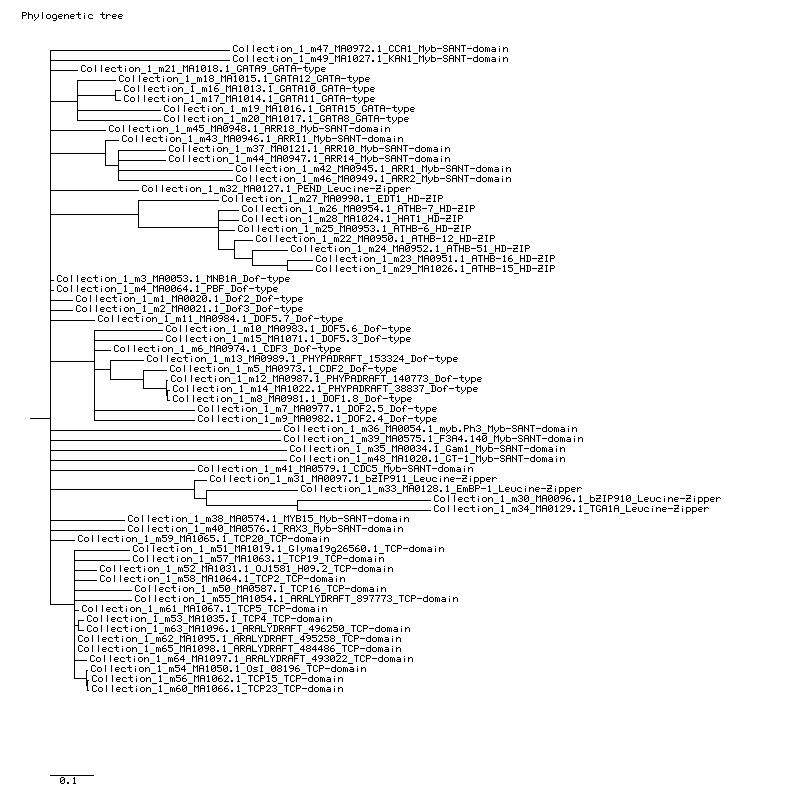


**Figure S6. Motif tree for the Plants dataset in Table S5.** The tree was generated by MOTIFSIM tool [13]. The tool achieves 97% correct classification of 65 motifs into their families.


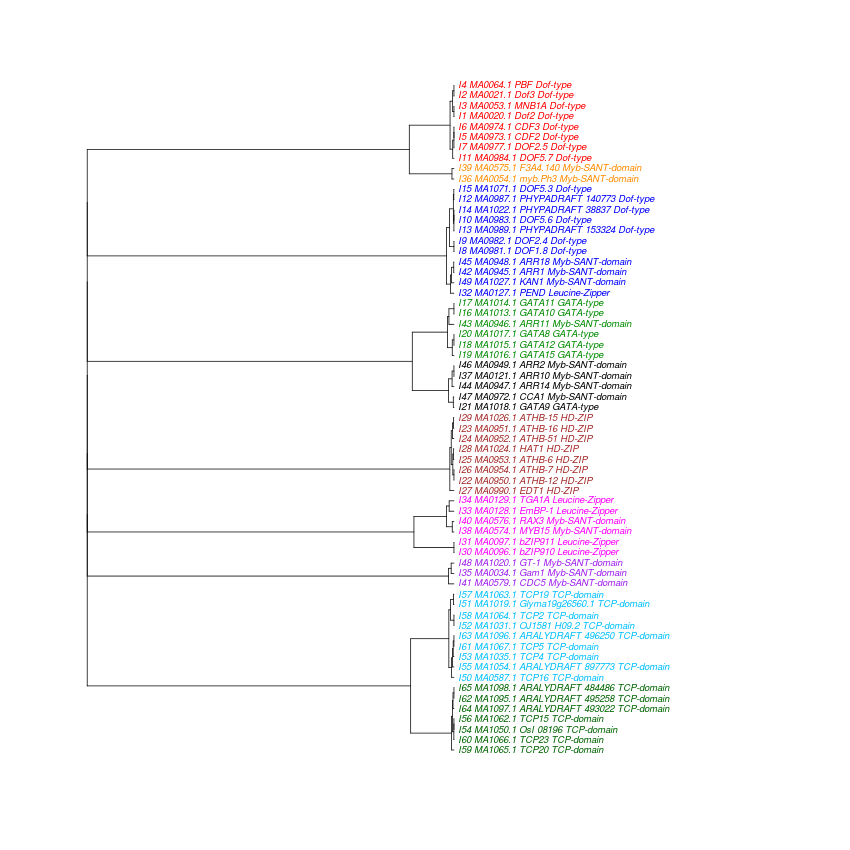


**Figure S7. Motif tree for the Vertebrates dataset in Table S6.** The tree was generated by Phylodendron for the result obtained by Matrix-clustering tool [11, 12]. The tool achieves 90% correct classification of 73 motifs into their families.


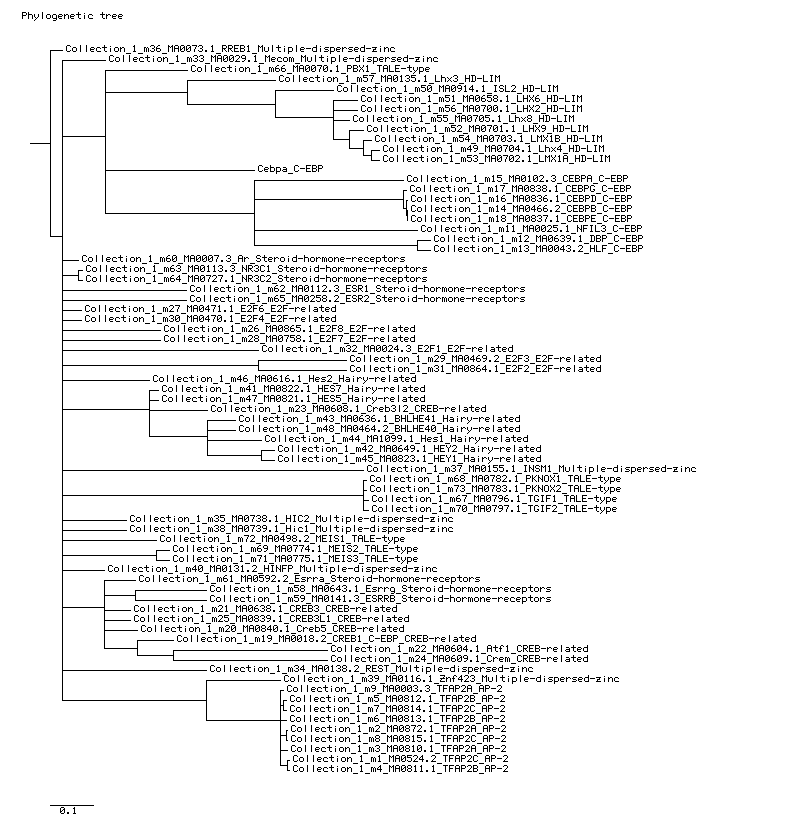


**Figure S8. Motif tree for the Vertebrates dataset in Table S6.** The tree was generated by MOTIFSIM tool [13]. The tool achieves 90% correct classification of 73 motifs into their families.


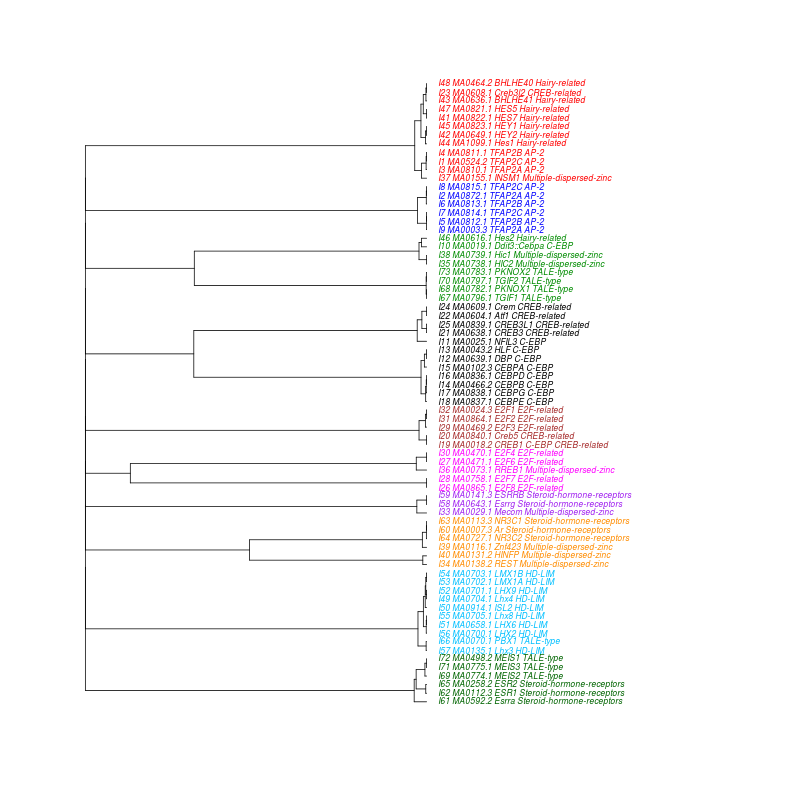


| **Figure S9. Performance comparison for six newer motif finders and MOTIFSIM on dataset *hm01g*. The selected global significant motif (best match for this dataset) from MOTIFSIM came from peak-motifs.** | 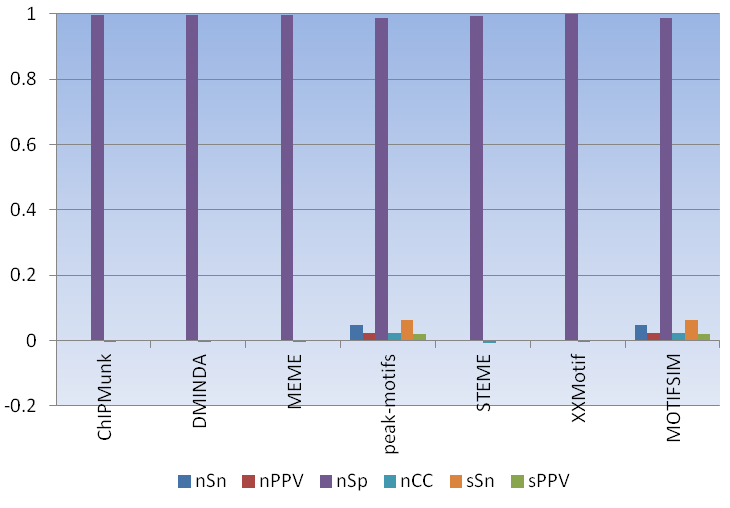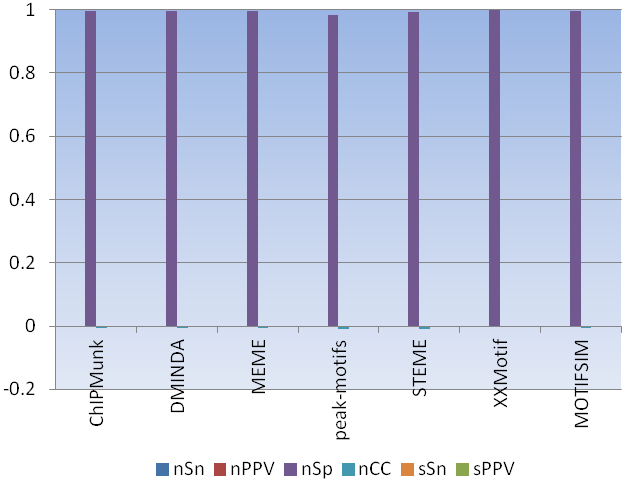  **Figure S10. Performance comparison for six newer motif finders and MOTIFSIM on dataset *hm04g*. The selected global significant motif (best match for this dataset) from MOTIFSIM came from DMINDA.** |
| --- | --- |
| 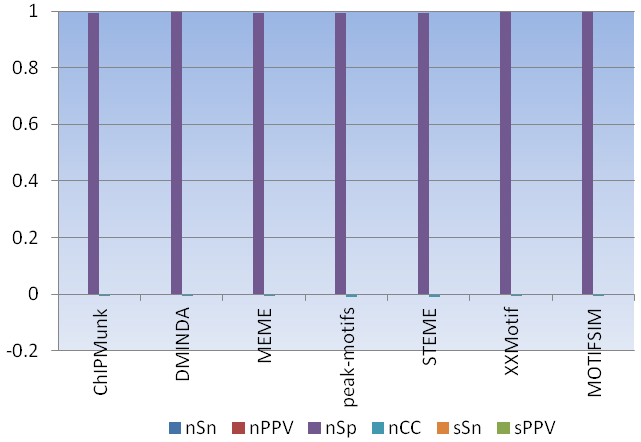  **Figure S11. Performance comparison for six newer motif finders and MOTIFSIM on dataset *hm15g*. The selected global significant motif (best match for this dataset) from MOTIFSIM came from XXMotif.** | 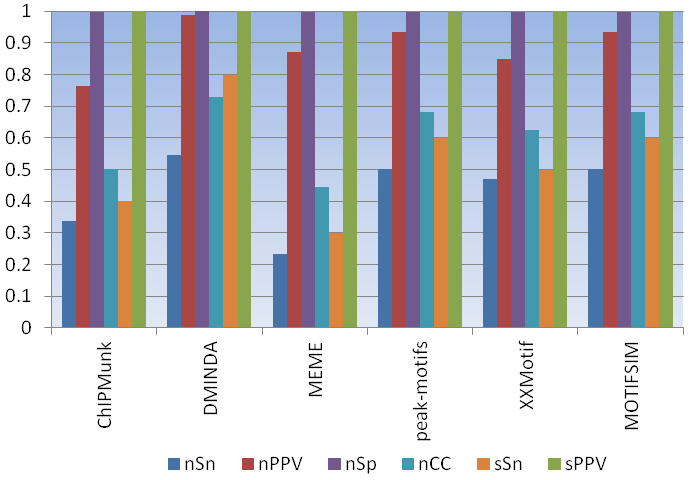  **Figure S12. Performance comparison for five newer motif finders and MOTIFSIM on dataset *hm17g*. The selected global significant motif (best match for this dataset) from MOTIFSIM came from peak-motifs.** |
| 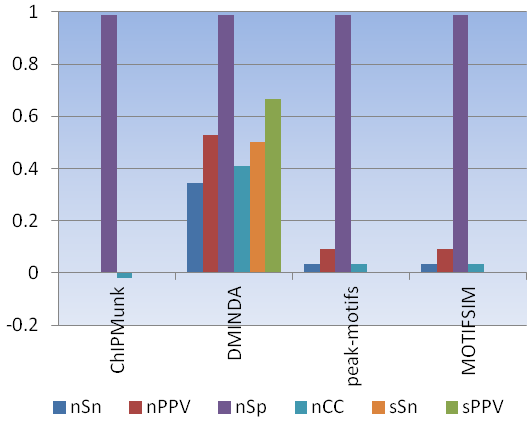  **Figure S13. Performance comparison for three newer motif finders and MOTIFSIM on dataset *hm19g*. The selected global significant motif (best match for this dataset) from MOTIFSIM came from peak-motifs.** | 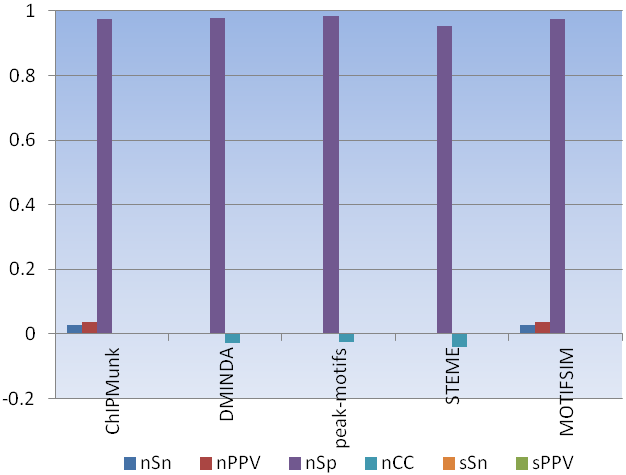  **Figure S14. Performance comparison for four newer motif finders and MOTIFSIM on dataset *hm22g*. The selected global significant motif (best match for this dataset) from MOTIFSIM came from ChIPMunk.** |
| 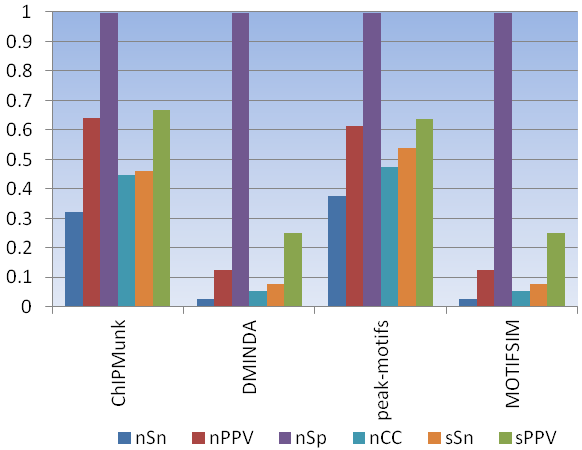  **Figure S15. Performance comparison for three newer motif finders and MOTIFSIM on dataset *yst09g*. The selected global significant motif (best match for this dataset) from MOTIFSIM came from DMINDA.** | 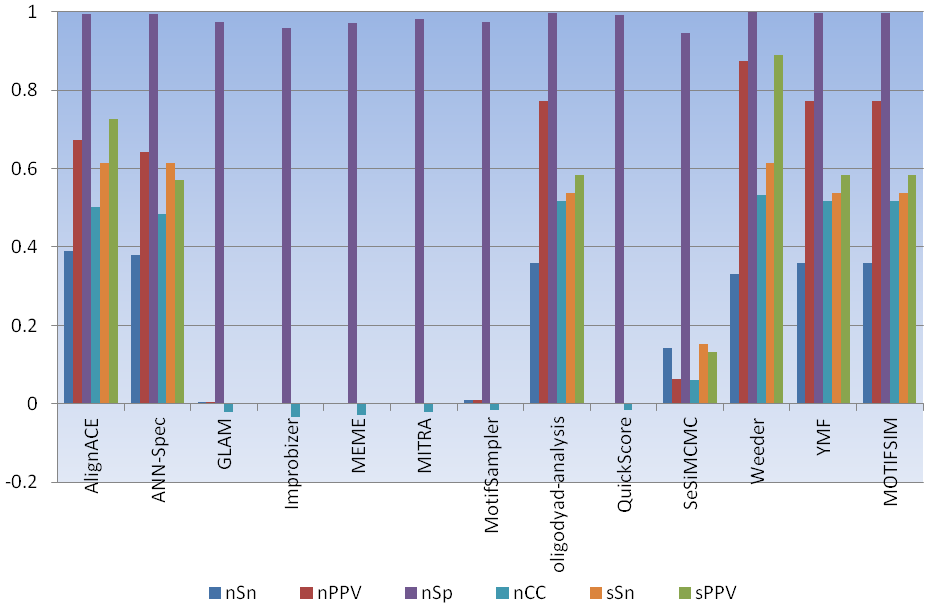  **Figure S16. Performance comparison for twelve older motif finders from Tompa *et al*. [2] and MOTIFSIM on dataset *hm08m*. The selected global significant motif (best match for this dataset) from MOTIFSIM came from YMF.** |
| 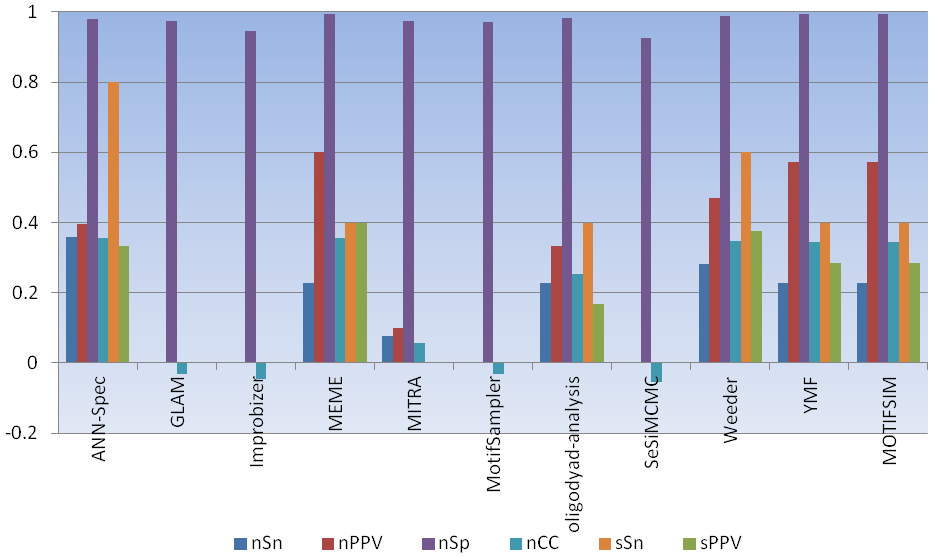  **Figure S17. Performance comparison for ten older motif finders from Tompa *et al*. [2] and MOTIFSIM on dataset *hm22m*. The selected global significant motif (best match for this dataset) from MOTIFSIM came from YMF.** | 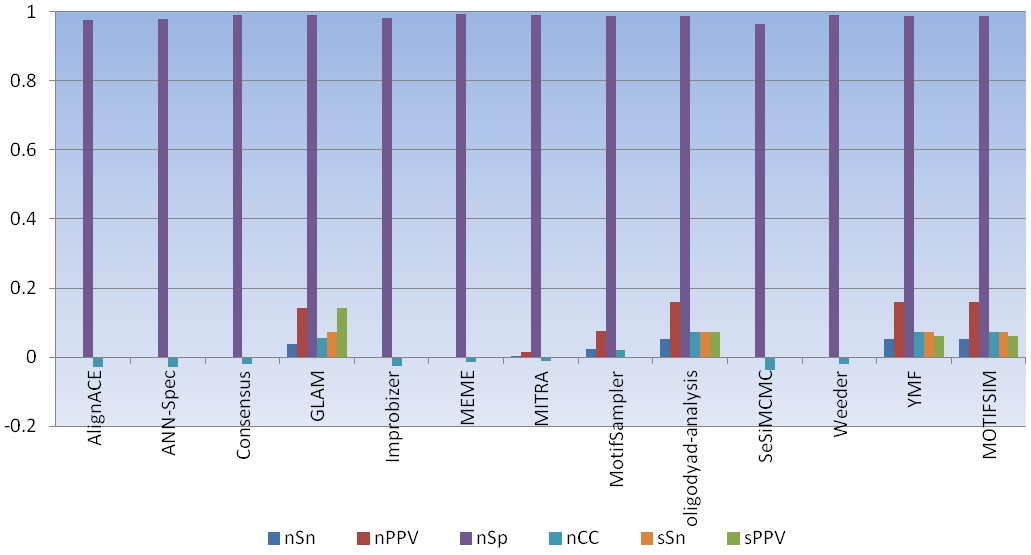  **Figure S18. Performance comparison for twelve older motif finders from Tompa *et al*. [2] and MOTIFSIM on dataset *mus04m*. The selected global significant motif (best match for this dataset) from MOTIFSIM came from YMF.** |
| 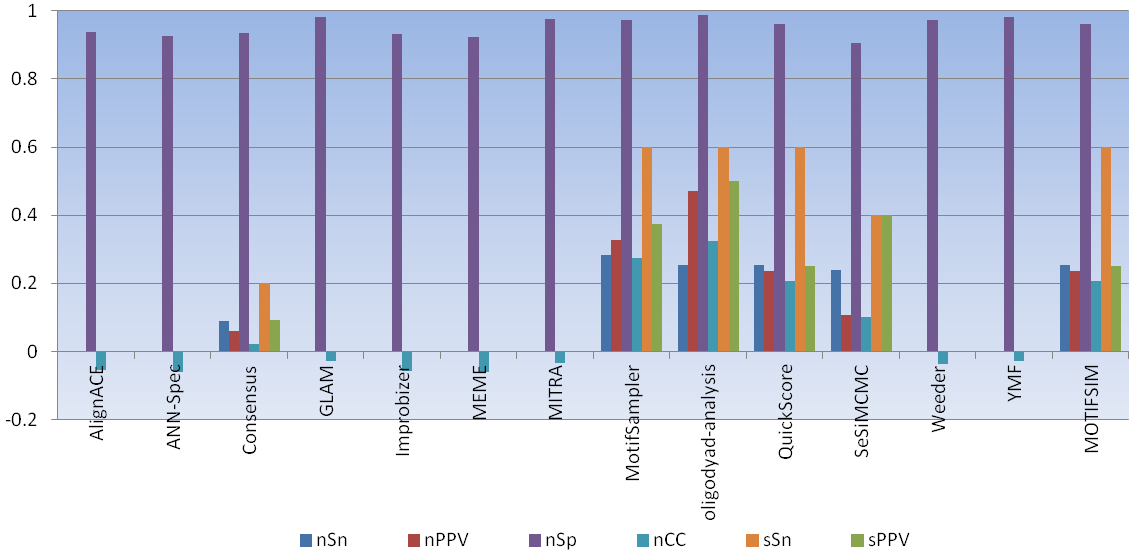  **Figure S19. Performance comparison for thirteen older motif finders from Tompa *et al*. [2] and MOTIFSIM on dataset *mus06g*. The selected global significant motif (best match for this dataset) from MOTIFSIM came from QuickScore.** | 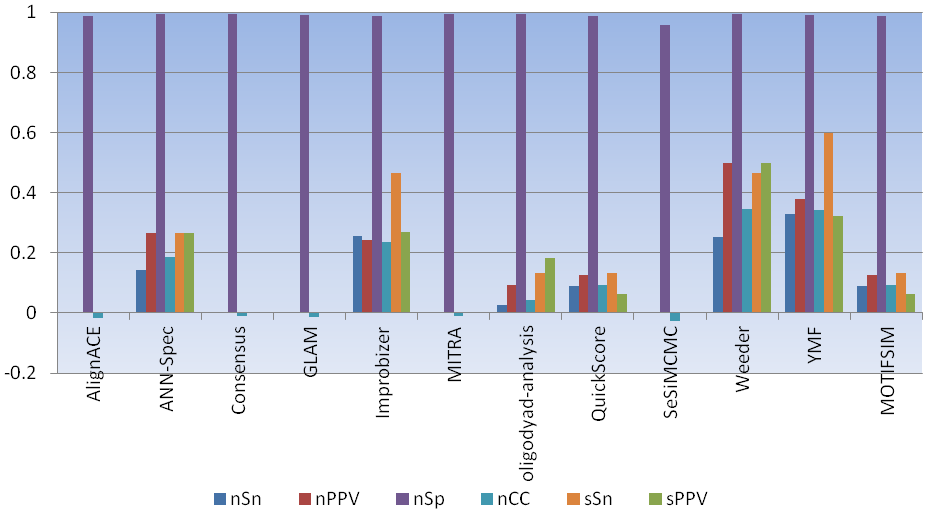  **Figure S20. Performance comparison for eleven older motif finders from Tompa *et al*. [2] and MOTIFSIM on dataset *mus10g*. The selected global significant motif (best match for this dataset) from MOTIFSIM came from QuickScore.** |
| 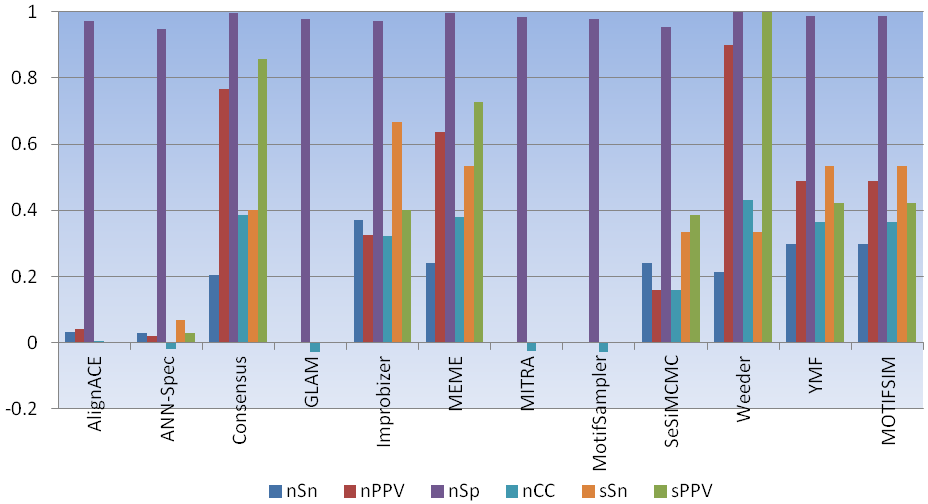  **Figure S21. Performance comparison for eleven older motif finders from Tompa *et al*. [2] and MOTIFSIM on dataset *mus11m*. The selected global significant motif (best match for this dataset) from MOTIFSIM came from YMF.** | 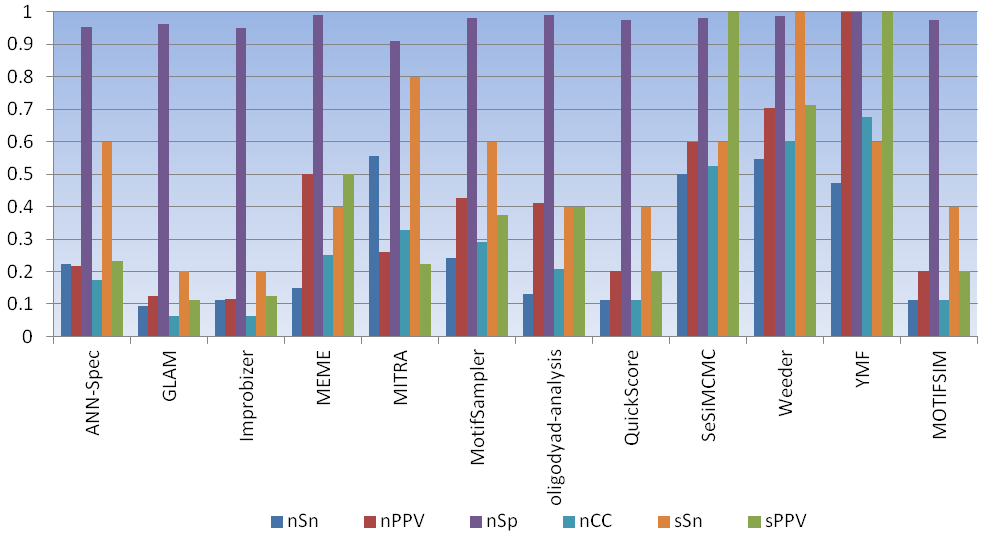  **Figure S22. Performance comparison for eleven older motif finders from Tompa *et al*. [2] and MOTIFSIM on dataset *yst02g*. The selected global significant motif (best match for this dataset) from MOTIFSIM came from QuickScore.** |
| 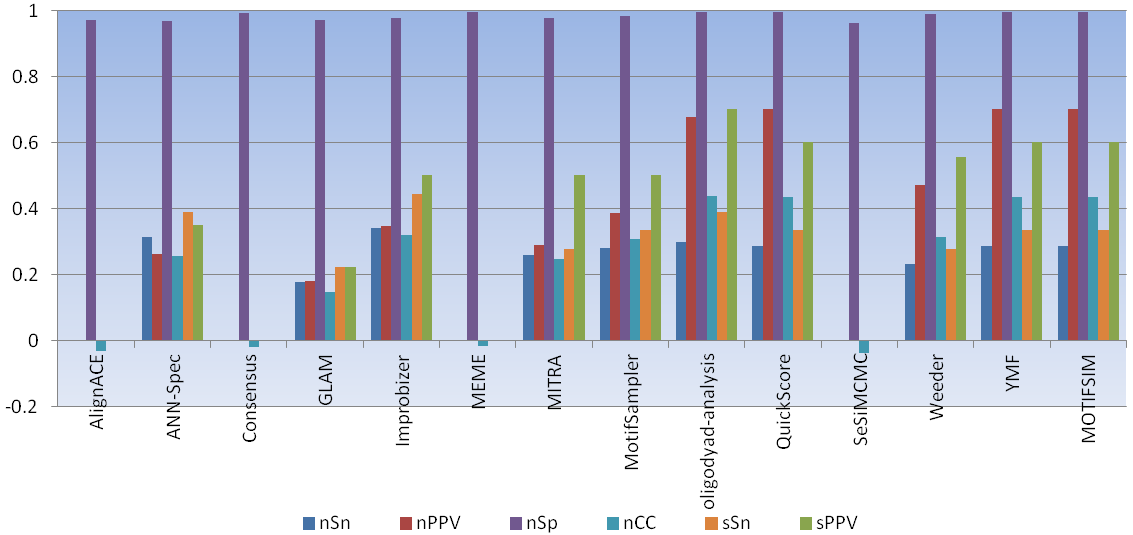  **Figure S23. Performance comparison for thirteen older motif finders from Tompa *et al*. [2] and MOTIFSIM on dataset *yst03m*. The selected global significant motif (best match for this dataset) from MOTIFSIM came from QuickScore.** | 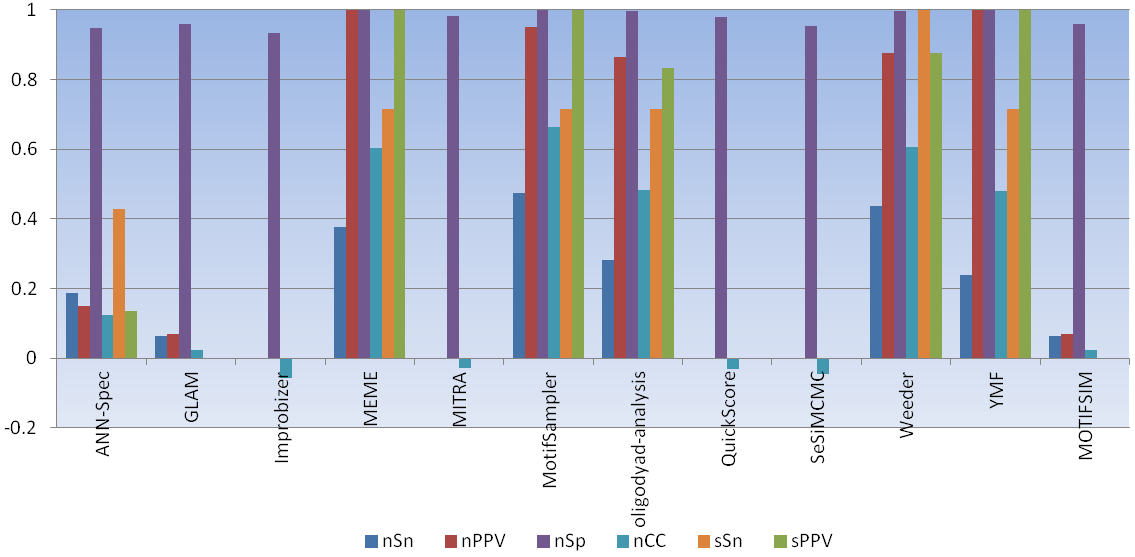  **Figure S24. Performance comparison for eleven older motif finders from Tompa *et al*. [2] and MOTIFSIM on dataset *yst06g*. The selected global significant motif (best match for this dataset) from MOTIFSIM came from GLAM.** |

**References**

1. Tran NTL, Huang C-H. MOTIFSIM: A Web Tool for Detecting Similarity in Multiple DNA Motif Datasets. BioTechniques. 2015;59(1):26-33.
2. Tompa M, Li N, Bailey TL, *et al*. Assessing computational tools for the discovery of transcription factor binding sites*.* Nat Biotechnol. 2005;23(1):137-144.
3. Kulakovskiy IV, Boeva VA, Favorov AV, *et al*. Deep and wide digging for binding motifs in ChIP-Seq data. Bioinformatics. 2010;26(20):2622-3.
4. Ma Q, Zhang H, Mao X, *et al*. DMINDA: an integrated web server for DNA motif identification and analyses. Nucleic Acids Res. 2014;42(Web Server issue):W12-9.
5. Bailey T, Williams N, Misleh C, Li W. MEME: discovering and analyzing DNA and protein sequence motifs. Nucleic Acids Res. 2006;34(Web Server issue):W369-W373.
6. Thomas-Chollier M, Herrmann C, Defrance M, Sand O, Thieffry D, van Helden J. RSAT peak-motifs: motif analysis in full-size ChIP-seq datasets. Nucleic Acids Res. 2012;40(4):e31.
7. Reid JE, Wernisch L. STEME: A Robust, Accurate Motif Finder for Large Data Sets. PLoS ONE. 2014;9(3):e90735.
8. Luehr S, Hartmann H, Söding J. The XXmotif web server for eXhaustive, weight matriX-based motif discovery in nucleotide sequences. Nucleic Acids Res. 2012;40(Web Server issue):W104-9.
9. Matys V, Fricke E, Geffers R, *et al.* TRANSFAC®: transcriptional regulation, from patterns to profiles. Nucleic Acids Res. 2003;31(1):374-8.
10. Mathelier A, Fornes O, Arenillas DJ, *et al*. JASPAR 2016: a major expansion and update of the open-access database of transcription factor binding profiles. Nucleic Acids Res. 2016;44(D1):D110-5.
11. Gilbert DG. Phylodendron 1999. http://iubio.bio.indiana.edu/treeapp/treeprint-form.html. Accessed 24 Jan 2018.
12. Castro-Mondragon JA, Jaeger S, Thieffry D, *et al*. RSAT matrix-clustering: dynamic exploration and redundancy reduction of transcription factor binding motif collections. Nucleic Acids Res. 2017;45(13):e119.
13. Tran NTL, Huang C.-H. MOTIFSIM 2.1: An Enhanced Software Platform for Detecting Similarity in Multiple DNA Motif Data Sets. J Comput Biol. 2017;24(9):895-905.
